# Removing bias against membrane proteins in interaction networks

**DOI:** 10.1186/1752-0509-5-169

**Published:** 2011-10-19

**Authors:** Glauber C Brito, David W Andrews

**Affiliations:** 1 Department of Biochemistry and Biomedical Sciences, McMaster University, Hamilton, Ontario L8N 3Z5, Canada

## Abstract

**Background:**

Cellular interaction networks can be used to analyze the effects on cell signaling and other functional consequences of perturbations to cellular physiology. Thus, several methods have been used to reconstitute interaction networks from multiple published datasets. However, the structure and performance of these networks depends on both the quality and the unbiased nature of the original data. Due to the inherent bias against membrane proteins in protein-protein interaction (PPI) data, interaction networks can be compromised particularly if they are to be used in conjunction with drug screening efforts, since most drug-targets are membrane proteins.

**Results:**

To overcome the experimental bias against PPIs involving membrane-associated proteins we used a probabilistic approach based on a hypergeometric distribution followed by logistic regression to simultaneously optimize the weights of different sources of interaction data. The resulting less biased genome-scale network constructed for the budding yeast *Saccharomyces cerevisiae *revealed that the starvation pathway is a distinct subnetwork of autophagy and retrieved a more integrated network of unfolded protein response genes. We also observed that the centrality-lethality rule depends on the content of membrane proteins in networks.

**Conclusions:**

We show here that the bias against membrane proteins can and should be corrected in order to have a better representation of the interactions and topological properties of protein interaction networks.

## Background

Membrane proteins are critical to diverse physiological functions and are directly implicated in many diseases. They represent one-third of the genome and 60% of the known drug targets [1]. Thus, it is important to be able to examine the cellular context in which membrane proteins interact with each other and with other cellular components, such as the components of signaling pathways. Functional gene networks including high quality membrane interactions would be particularly useful to probe crosstalk between signaling pathways and thereby improve our understanding of how proteins function synergistically and antagonistically to control cellular phenotypes. By providing the context for cellular processes needed to interpret the results, network modeling is also important to understand how genes modulate the activity of drugs and reagents [2] and in high throughput screening projects using shRNAs, siRNAs, deletion libraries, etc. [3-5]. Networks have been used most successfully to provide insights into the functions of the proteins encoded by the yeast genome [6-10], since the most complete and best annotated publicly available gene interactions are available for this widely used model organism (BIOGRID database, version 2.0.57).

In probabilistic network models, statistical associations established for pairs of genes are used to generate a network in which the genes likely to participate in the same cellular pathway or process are connected by probabilistic functions. Each connection in the network is scored with the likelihood of the linked genes belonging to the same pathway. This results in a probabilistic view of interacting genes in which, instead of being defined as 'interacting' or 'non-interacting', each potentially interacting pair is placed in a graded spectrum of confidence levels.

Predictions performed using an interaction network are typically based on the assumption that the experimental data is non-biased, which is not always the case [8]. Because the assumption is that interactions that are present multiple times are more reliable (given higher weight) a bias is introduced due to over-representation by popular methods for PPI identification, such as the yeast two-hybrid system (Y2H) [11,12] and affinity capture-mass spectrometry (affinity capture-MS) [13,14]. The Y2H method requires that the two proteins localize to the nucleus; however, integral membrane proteins expressed in an aqueous nuclear environment may aggregate or misfold [11,12,15], resulting in depletion of these proteins from the final interaction dataset. Also, the datasets derived from affinity capture-MS can be biased against integral membrane proteins, because biochemical purifications require detergents to isolate proteins away from the lipid bilayer [16]. In addition, it is impossible to optimize the solubilization conditions for all membrane proteins and complexes due to the large-scale nature of these projects [17,18]. Furthermore, membrane proteins tend to be expressed at a lower level compared to soluble proteins [19], and affinity capture-MS preferentially detects abundant proteins [8,20]. The potential under-representation of membrane proteins can have important effects on the computational prediction of the localization and function of gene products due to underestimation of reliability. Considering that genomic studies suggest that membrane proteins make up ~25% to ~33% of the predicted proteins in an organism [6], network bias may help to explain why many disagreements occur when predicting the localization of proteins belonging to the secretory pathway [21].

Recently, some approaches have measured interactions between proteins in their natural cellular context, diminishing the bias against membrane proteins. Tarassov *et al. *[16] have performed a systematic binary screen for PPIs in yeast using protein-fragment complementation assays (hereafter PF-PCA) based on murine dihydrofolate reductase. This method provides an alternative approach to detect PPIs but the PPIs observed show enrichment in proteins from organelle membranes when compared to the whole genome [16] suggesting a bias in favor of membrane proteins. Similarly, Miller *et al*. [22] used a modified split ubiquitin yeast two-hybrid technique (hereafter SU-2HY) specifically designed to increase the representation of integral membrane proteins, and observed 1,985 putative interactions involving 536 membrane-associated proteins. This screen was specifically directed at membrane protein interactions and has identified large numbers of interactors for some membrane proteins that were not seen using other techniques.

Genetic interactions tend to be less biased against membrane proteins although the interpretation of genetic interactions in a physical cellular context is a major biological challenge. Nevertheless for any given gene significant correlation between the genetic and physical interaction degree has been observed [4]. Furthermore, significant overlap between genetic interactions and PPI pairs (10 to 20%) was also reported by these authors, thus justifying the utility of genetic interactions for inferring functional associations between genes. One additional advantage of including genetic interactions in a probabilistic network is that PPIs connect relatively fewer bioprocesses than genetic interactions. Thus, physical interactions are highly informative of local pathway architecture but provide a less complete picture of functional modules or interconnections between them [4]. Therefore, to optimally contextualize genes, functional networks should integrate many sources of evidence of interaction among proteins and genes. In this way, the transfer of function annotation occurs from one gene to another via biological relationships that include information such as correlated gene expression [23], correlated phylogenetic profiles [24], PPIs [25], and phenotyping experiments [26].

To use gene network neighbors to predict new annotations for genes, such as function and localization, requires a high-confidence network. In this study a classifier was trained using Gene Ontology as a gold-standard set of annotations and validated with an independent dataset of localization annotations. We provide proof-of-principle evidence that genes linked in a probabilistic network constructed with increased coverage of membrane proteins can give rise to important insights in membrane biology. This approach provides a rational and quantitative foundation to analyze and visualize relationships involving membrane proteins, and results in a different view of the yeast membrane interactome, particularly as it applies to the centrality-lethality rule.

## Results

### The bias against membrane proteins

**Figure 1:**
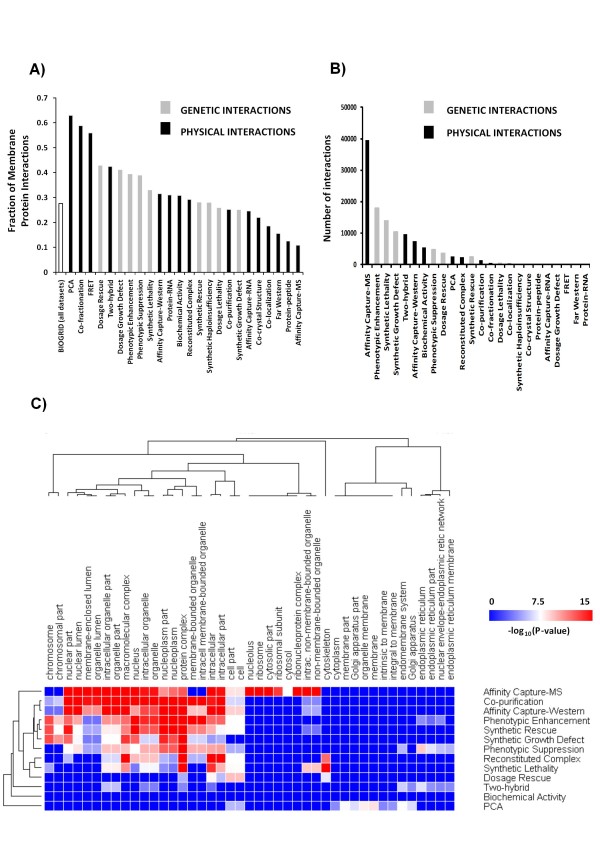
**Interactions involving membranes are under-represented in the BIOGRID database**. A) Fraction of the interactions identified using the method specified (below the bars) that involve at least one membrane protein. B) The total number of interactions reported by the method specified. C) Identification of ontology defined cell compartments that are over-represented by different methods for capturing interaction data. The extent of over-representation for different ontology defined cell compartments was quantified using BINGO for the specified methods. The cell compartments and methods were hierarchically clustered according to enrichment (P-values). Many of the over-represented compartments (red) relate to nuclear localization.

To evaluate the coverage of interactions involving membrane proteins we performed an analysis with all 160,613 yeast genetic and physical interactions present in the BIOGRID database (version 2.0.57). We divided the interactions according to the method used to generate them, and counted the number of interactions involving at least one membrane protein (GO:0016020 and its children GO Terms). We observed a wide variation in both genetic and physical membrane protein interactions (63%-11%) depending on the method used to generate the data (Figure 1A). As expected, the affinity capture-MS method showed the highest bias against interactions involving at least one membrane protein. In addition, a GO enrichment analysis with 1000 interactions randomly selected from this same dataset showed an under-representation of the GO categories "membrane", "mitochondrial membrane", and "plasma membrane", with p-values, after Bonferroni's correction, of 10^-15^, 10^-3^, and 10^-2^, respectively. The bias observed with the affinity capture-MS method has a significant impact on the total coverage of membrane proteins, since this method contributes more than 25% (~40,000 interactions) of the total interactions in the BIOGRID database (Figure 1B), although it has only ~10% of its interactions involving a membrane protein (Figure 1A). The method with the highest density of membrane PPIs was the protein complementation analysis (PCA), but it represents only about 2% of the total interactions.

When a global analysis of GO enrichment was performed to identify potential bias in the BIOGRID database, we observed an under-representation of interactions involving categories related to "mitochondrion" and "membrane" (Table 1), a result due in part to the predominance of affinity capture-MS data. We interpret the under-representation of mitochondria proteins as a result of the high fraction of membrane proteins present in this organelle (42%, 479/1133). In contrast, the analysis of over-represented GO categories revealed that proteins associated with complexes and proteins located in the nucleus are over-represented, with 77% and 47% more interactions involving these sets of proteins than what would be expected by chance, respectively (see Table 1 for enrichment p-values). These categories are also highly enriched in the affinity capture-MS dataset (Figure 1C).

**Table 1:** Analysis of cell compartment enrichment in the BIOGRID database.

GO ID	GO Term	Median p-value
**Overrepresented categories**:		

32991	macromolecular complex	4.96E-105

43234	protein complex	1.80E-78

44428	nuclear part	1.06E-66

5622	intracellular	2.74E-54

44424	intracellular part	3.72E-53

44422	organelle part	8.61E-51

44446	intracellular organelle part	8.61E-51

5634	nucleus	2.43E-52

43229	intracellular organelle	8.85E-48

43226	organelle	8.85E-48

31981	nuclear lumen	1.27E-53

44451	nucleoplasm part	5.98E-38

5654	nucleoplasm	1.75E-38

44464	cell part	3.54E-33

5623	cell	4.46E-33

43228	non-membrane-bounded organelle	1.67E-34

43232	intracellular non-membrane-bounded organelle	1.67E-34

43233	organelle lumen	1.02E-31

31974	membrane-enclosed lumen	1.02E-31

43231	intracellular membrane-bounded organelle	4.99E-31

43227	membrane-bounded organelle	4.99E-31

16591	DNA-directed RNA polymerase II, holoenzyme	4.98E-11

5694	chromosome	7.67E-15

30529	ribonucleoprotein complex	2.76E-12

5730	nucleolus	6.52E-13

502	proteasome complex	2.37E-06

44427	chromosomal part	1.61E-14

5856	cytoskeleton	3.25E-07

5679	chromatin remodeling complex	1.70E-09

123	histone acetyltransferase complex	5.87E-10

44430	cytoskeletal part	1.65E-05

22624	proteasome accessory complex	9.47E-04

5838	proteasome regulatory particle	9.47E-04

44452	nucleolar part	2.30E-08

5669	transcription factor TFIID complex	7.26E-03

5681	spliceosome	4.85E-04

119	mediator complex	3.91E-04

124	SAGA complex	1.04E-05

5667	transcription factor complex	3.06E-04

812	SWR1 complex	1.15E-03

44453	nuclear membrane part	6.00E-03

**Underrepresented categories**:		

943	retrotransposon nucleocapsid	2.88E-05

31224	intrinsic to membrane	1.22E-05

44429	mitochondrial part	3.18E-04

5740	mitochondrial envelope	4.89E-03

16021	integral to membrane	7.67E-05

44425	membrane part	1.85E-03

5743	mitochondrial inner membrane	7.18E-03

5739	mitochondrion	6.34E-03

The median p-values were calculated after sampling 10 random sets of 1000 interactions from BIOGRID. P-values are shown after Bonferroni's correction for multiple hypotheses.

**Figure 2:**
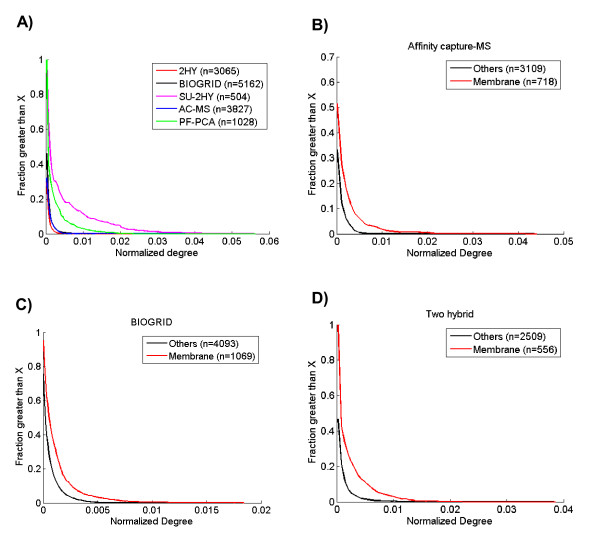
**Membrane associated interactions have a higher normalized node degree than other nodes in yeast**. A) Comparison of the cumulative distribution of normalized degree for membrane associated and other nodes from different datasets. Datasets with a higher proportion of membrane associated interactions (PF-PCA and SU-2HY) have higher normalized node degree. B-D) Comparison of membrane nodes with other nodes within single datasets B) Affinity capture-MS, C) BIOGRID, D) Two hybrid used to generate interaction data. 2HY, Two hybrid; BIOGRID, raw data from BIOGRID; SU-2HY, Split-ubiquitin two hybrid; AC-MS, Affinity capture-mass spectrometry; PF-PCA, protein-fragment complementation assay. Between parentheses is the number of nodes.

The assumption underlying our interpretation of this data which led to our hypothesis of bias against membrane proteins is that membrane proteins engage in at least the same number of true interactions as other protein classes. This assumption is based in part on the extensive literature suggesting that membrane proteins function in complexes, which are a good proxy for interacting proteins. Moreover, we investigated this directly by evaluating the distribution of interaction degree normalized by the total number of interactions in datasets rich in membrane interactions (e.g., PF-PCA and SU-2HY, both with more than 65% membrane interactions) or with a poor coverage of membrane interactions (e.g., two hybrid (2HY), (29%) and affinity capture-MS (14%)). We normalized the node degree in order to make the different datasets more comparable since the average degree is dependent on the number of interactions and nodes identified by the method. By normalizing by the total number of interactions, we assume the methods detect the same relative number of nodes, independently of localization (membrane, nucleus, cytoplasm, etc). Thus, our results are consistent with the hypothesis that membrane proteins undergo at least as many interactions as other proteins, since we observed that datasets rich in membrane interactions tend to have a higher normalized degree (Figure 2A) than the datasets with fewer of these interactions.

In order to exclude potential bias from the interaction detection method used, we also evaluated the normalized degree for membrane and non-membrane proteins within datasets (Figure 2B-D). Again, we observed that membrane proteins have a higher normalized node degree than other proteins, suggesting the correctness of our assumption that membrane proteins have at least the same number of protein-protein interactions as other protein classes. For example, 52% of membrane proteins have a normalized degree higher than 0.005 in the two-hybrid dataset, while only 12% of other proteins have a degree higher than that (Figure 2D).

Naturally, physically interacting proteins must co-occur spatially and temporally and any network constructed must consider that the interaction datasets available are highly biased to specific locations. We consider this location bias as inevitable, and here we apply a new approach to minimize its adverse effects for membrane network reconstruction.

### The Probabilistic Interaction Network (PIN)

As a consequence of the bias discussed above, a probabilistic network connecting many different types of biological information between genes and proteins can have as its main caveat the different properties of the original datasets. To integrate heterogeneous datasets it is necessary to consider the tradeoff between coverage and prediction performance. The simple union of networks results high coverage but low prediction performance given the quality of some datasets. One alternative method is to consider the intersection among the datasets, which results in good prediction performance, but the coverage is low [27]. To overcome these limitations, we applied a probabilistic approach to construct a network less biased against membrane proteins. We used a simple and general error model (based on the hypergeometric distribution) to calculate the probability for each interaction occurring at random, followed by logistic regression to optimize the network performance for interactions involving membrane proteins by varying the weights of each network. In this way the network weights were simultaneously optimized for best prediction performance on the training set, and the resulting logistic regression classifier was evaluated using the test set.

Hypergeometric scoring has proven to be robust across different settings [8,28-32]. It also penalizes highly connected "promiscuous" interactors, since the probability of occurrence of an interaction with this type of partner is high in a random network resulting in a proportionally low score in the network. Hypergeometric scoring favors interactions between rare proteins over those between common proteins, because to be predicted to interact, common proteins must be virtually always observed as interacting pairs [28].

**Figure 3:**
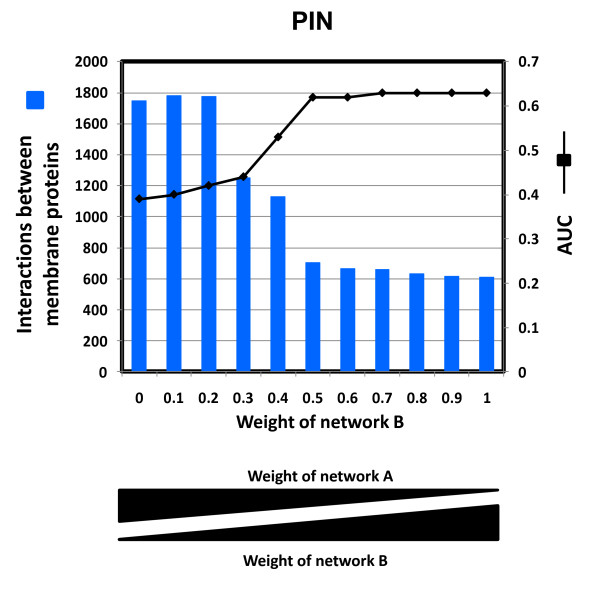
**Optimization of the weighting of the two networks in PIN**. A) PIN was constructed by the optimized combination of two networks: A (enriched in membrane interactions) and B (the rest of BIOGRID). The Area Under Curve (AUC) of Precision-Recall plots for the top 5,000 interactions was used for selecting the optimum weight for network B using membrane proteins from GO Slim as an established benchmark dataset. The AUC was calculated for different weights for network B, and the weight corresponding to highest prediction performance and higher number of membrane interactions (AUC = 0.62) was chosen (weight of network B = 0.5). The increased coverage of membrane interactions reflected as the weight of network A (B) in the logistic regression is increased (decreased).

Here we combined the hypergeometric scoring with logistic regression to construct a network with less bias against membrane interactions. This was achieved by the following steps:

1) As mentioned earlier, although data from PPIs is usually biased against membrane proteins, some methods (PF-PCA and SU-2HY) have produced data with a good coverage of membrane protein interactions [16,22]. We applied the hypergeometric distribution model to construct two networks: network A, constructed based on interactions data from datasets PF-PCA and SU-2HY, has 4,452 interactions, and network B, constructed with BIOGRID data excluding these two datasets. Network A is highly enriched in interactions involving membrane proteins (corrected p-value = 10^-70^).

2) We generated 10 networks using different weights of network A and B, which were combined by a simple logistic regression model with one free parameter (Figure 3) [31].

3) Then we used the guilt-by-association approach to select the best network from step 2. Our guilt-by-association approach works by transferring annotation from one gene to another via gene interactions. Thus, a specific localization may be assigned to a gene based on the profile of its neighbors. We generated a functional linkage network with edge weights reflecting the probability that two genes co-localize (same GO Slim term); for that, we used the networks from step 2 to assign a score to each combination (candidate gene, GO Slim term) based on the weights of interactions between the candidate gene and genes currently assigned to that GO term. For example, if a gene is connected to several genes located in the membrane, this gene gets a higher score (for membrane localization) than other gene that is not connected to membrane genes.

4) We applied the guilt-by-association approach to optimally select one of the 10 networks generated in step 2. For that, a random set of 2/3 and 1/3 of the genes in GO Slim were selected as training and test sets, respectively. In the end, we selected the network with the weights that showed the best crossvalidation performance using AUC of precision versus recall curve and using membrane genes as reference.

Thus, we used two measurements for optimality criteria: 1) The area under the precision versus recall curve for a random set of 2/3 and 1/3 of the membrane genes in GO Slim selected as training and test sets, respectively and 2) the increased coverage of membrane interactions reflected by the weight of network A in the logistic regression. The weight we chose as optimal for constructing the probabilistic interaction network (PIN) was 0.5 and corresponds to the point at which the area under the curve reaches a *plateau *(Figure 3 and Additional file 1). Beyond this point the variation is very small, (from 0.62 to 0.63) and unlikely to be significant. We selected this point as optimal within the *plateau *as it resulted in the highest coverage of membrane proteins. Please, see Methods for more details.

The rapid change in area under the curve observed around the weights 0.3-0.5 is likely the result of the tradeoff between membrane coverage and prediction performance in our model. This suggests that network A, while rich in membrane interactions, does not have as much prediction performance as the other BIOGRID interactions combined. Overall prediction performance follows the relative weight of the networks A and B and the abrupt change identifies the point at which adding additional weight to the interactions in network A begins to decrease the overall prediction performance unacceptably. We observed good coverage of network A in PIN (Additional file 2, Figure S1) with 828 interactions retained from network A in the top 5000 interactions. Thus, by optimizing the combination of these two networks we obtained a good representation of membrane interactions while maintaining a good prediction performance.

The reason we selected the PPI datasets SU-2HY and PF-PCA as network A was because these datasets showed high quality and high coverage of membrane interactions. To demonstrate the value of using selected high quality datasets we constructed a second network where instead of using a membrane-rich interaction dataset we used a PPI network from iREF database as network A'. This iREF network does not contain data used to construct PIN, has 9,412 interactions and 16.2% of its interactions involve at least one membrane protein. The membrane network used to construct PIN (network A) has fewer interactions but 71.3% of them are membrane interactions. Following the same approach as we applied to PIN, we plotted the area under the curve and membrane interaction content versus the weight of the iREF network (Additional file 2, Figure S2). As above, the weight selected (0.8) represents the beginning of the plateau of area under the curve (0.38) with the highest coverage of membrane interactions (657 interactions). For comparison, the weight selected for network A in PIN (0.5) resulted in a network of 706 membrane interactions and area under the curve of 0.62. Thus, PIN has ~7% (706/657) more membrane interactions with 63% higher area under the curve (0.62/0.38). These results suggest that the performance of our approach depends on the fraction and accuracy of membrane interactions in datasets used to construct the network. This performance and coverage of membrane interactions could not be reached by using the hypergeometric model alone. As shown below, PIN's performance was superior to the hypergeometric model in all of the evaluations we performed.

To provide additional information on the network properties we used the top 5000 interactions to evaluate the network density of PIN compared to the hypergeometric model alone. The network density is the number of connections present out of all possible connections [33]. PIN produced a network with 2999 nodes (network density = 0.001) in the main network. Using only the hypergeometric model resulted in a network with the similar density but fewer nodes (2723).

### Performance evaluation of PIN

**Figure 4:**
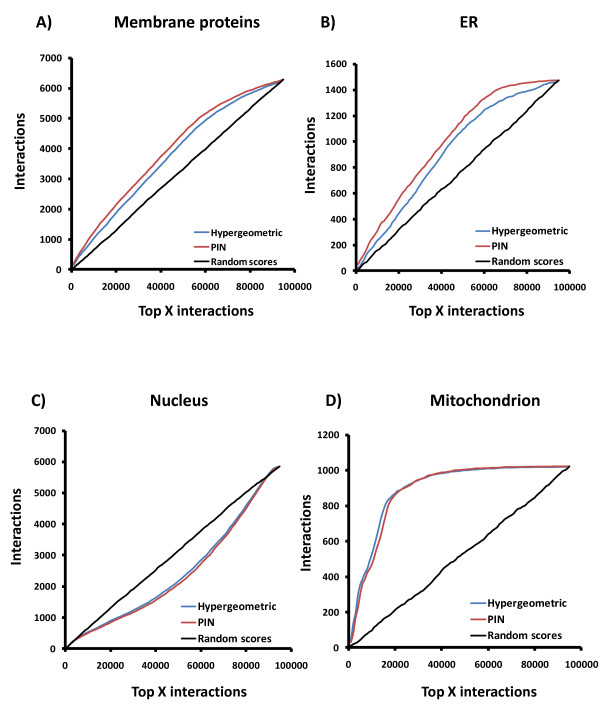
**PIN increases the proportion of membrane associated interactions in the network compared to a hypergeometric model or random scoring**. Coverage of interactions by PIN in different cell compartments: A) Membranes, All membranes; B) ER, endoplasmic reticulum; C) Nucleus; D) Mitochondrion.

To analyze the performance of our PIN, we used a publicly available subcellular localization dataset generated from a yeast strain collection expressing full-length proteins tagged at the carboxyl-terminus with green fluorescent protein [34]. While the bias observed in this dataset is lower than techniques such as mass spectrometry, it is still present (e.g. tail-anchored membrane proteins are expected to be under-represented). Nevertheless, interactions involving two genes whose proteins localize in the same compartment are more likely to be authentic than interactions between proteins located in different subcellular compartments. Thus, the interactions in the PIN were ranked in decreasing order of statistical significance and for each cutoff (window size = 1000 interactions), we counted the number of interactions involving genes annotated as located in the same compartment. As expected, we observed a relative increase in coverage of membrane proteins when using PIN (Figure 4A). We also observed a higher fraction of interactions from the same compartment in comparison with the hypergeometric model for compartments related to membrane especially the endoplasmic reticulum (ER) (Figure 4B). These interactions are likely to be primarily interactions that represent the traffic of proteins between organelles [16]. Furthermore, the PIN and the hypergeometric model performed similarly for nuclear proteins and mitochondria (Figures 4C and 4D), indicating that the PIN is not biased against these proteins.

Nuclear proteins showed an inverted behavior: a decrease in interaction density both using PIN and the hypergeometric model. We interpret this last result partially as a consequence of both procedures minimizing the effects of nuclear contaminants (false positives) that bind to DNA (transcription factors) or RNA (binding factors). These contaminants are usually co-purified with DNA or RNA, thus forming a bridge with other proteins. Because they are common contaminants, these indirect interactions are penalized under both the PIN and the hypergeometric model [35]. As a result, when the PIN was divided in quintiles of significance, an analysis of the cellular component of the Gene Ontology showed a significant enrichment of nuclear proteins mostly in the 4^th^-5^th^ quintiles (lowest statistical significance) (Additional file 2, Figure S3-A). Similarly only low significance interactions showed enrichment in biological processes and molecular functions that typically involve protein binding to DNA or RNA (Additional file 2, Figures S3-B and C).

The hypergeometric model performed similarly to PIN, with an increased number of interactions compared to the random network scores for both mitochondria (Figure 4-D) and cytoplasm proteins (not shown). The reason the two networks showed an accentuated increase of interactions in mitochondria may be because 42% of mitochondrial proteins are located in the membrane, which represents a high fraction of the total mitochondria proteins. As expected for a high confidence interaction network and consistent with the PIN removing the bias against membrane proteins, rather than introducing a bias towards them, minimal enrichment in Gene Ontology terms was observed in the 1^st^ quintile of significance (Additional file 2, Figure S3 A-C).

In our present study, we removed the bias against membrane proteins by increasing the relative contribution from a small number of datasets with high quality membrane protein data. As a result, there would be an unavoidable increase in false positive interactions for lower significance scores as discussed above. To examine this issue further we examined our PIN for over- and under-representation of GO categories as evidence of bias. Assuming that these proteins, e.g. proteins from complexes, have the same average number of interactions as other protein classes, there should not be any enrichment of these classes in the PIN compared to the whole genome since the technique would ideally reflect the natural composition of the interactome. Most GO categories were not highly over-represented in quintiles lower than 4^th^ (Additional file 2, Figure S3 A-C). For example, protein complex only begins to be over-represented in 3^rd^ quintile. Taken together these results suggest that our model successfully compensated for the pre-existing bias against membrane proteins in the network, resulting in a more representative collection of real interactions that are not connected to other unrelated GO categories.

We also downloaded from Gene Ontology a list of 48 proteins from membrane complexes in yeast. Then, we counted the number of interactions involving two proteins from this list (Additional file 2, Figure S4). As expected, we observed that PIN showed a higher coverage of interactions for these complexes than the hypergeometric model or BIOGRID raw data. We also evaluated the coverage of 1223 transport genes (GO: 0006810) using the same approach. Again, the coverage of interactions involving transport genes was higher using PIN than the other two networks.

As another approach to validate PIN, we characterized network performance using a positive reference set (PRS) and a random reference set (RRS) for protein interactions [36,37]. The PRS set contained 9,412 unique interactions not previously used in our analysis and was downloaded from iRefIndex database, which consolidates protein interactions from 10 databases [38]. This reference set was generated by selecting those interaction datasets present in the iRefIndex database but not in the BIOGRID database used to construct PIN. These datasets were indexed by the PubMed ID in order to select inedit data as our PRS. For RRS, 100,000 unique random interactions were generated with the same node degree distribution in PRS from a list of all genes present in BIOGRID. Because there is no available gold standard for non-interacting proteins and because randomly chosen protein pairs are unlikely a priori to interact, our RRS serves as a reasonable negative control set [37].

**Figure 5:**
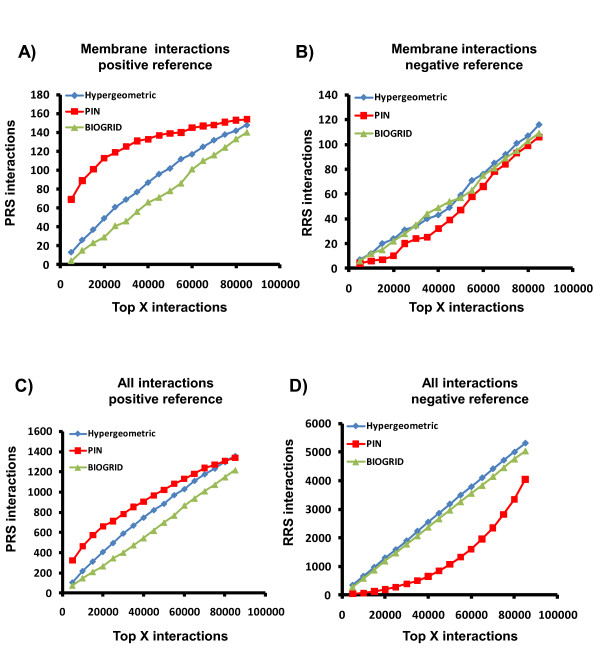
**PIN provides higher coverage of authentic interactions and increased rejection of spurious interactions**. A) PIN (RED) permits Identification of more positive reference set (PRS) membrane interactions than the Hypergeometric (blue) and BIOGRID (green) networks. B) PIN rejects Identification of slightly more random reference set (RRS) membrane interactions than the Hypergeometric and BIOGRID networks. C) PIN permits Identification of more positive reference set (PRS) interactions than the Hypergeometric model and BIOGRID networks. D) PIN rejects identification of more random reference set (RRS) interactions than the Hypergeometric and BIOGRID networks.

Comparing the performance of PIN with the hypergeometric model or BIOGRID raw data for the detection of PRS (Figure 5A and 5C) or RRS (Figure 5B and 5D) interactions revealed that more PRS interactions were detected using PIN than with the other datasets. We also observed that the increased detection of interactions in PRS pairs by PIN did not correspond to a high relative number of positive-scoring RRS pairs (false positives) for membrane interactions (Figure 5B). In contrast, the hypergeometric model, showed an increased sensitivity at the expense of selectivity since it detected slightly more membrane interactions using RRS (higher false positive rate) than the BIOGRID raw data (Figure 5D).

Even though PIN was optimized for the detection of membrane interactions it showed a higher coverage of PRS (higher true positive rate) than the hypergeometric model for all genes (Figure 5C) and even nuclear proteins (not shown) without increasing coverage of RRS (Figure 5D). It is likely that by increasing the coverage of bona fide membrane interactions, the other interactions in the lower quintiles were the higher confidence interactions from BIOGRID. Thus, decreasing the bias against membrane proteins increased the proportion of true interactions in the high confidence quintiles independently of their localization.

### Network properties

Our PIN allows an in-depth analysis of membrane proteins in the context of their network surroundings. The spatial properties of the network that are preserved under continuous deformations of interactions connecting surrounding nodes - topological properties - can only be analyzed at the network level, as genes/proteins by themselves cannot provide full information about the robustness of cellular function [39]. Thus, we assessed the network topologies for the PIN with the aim of uncovering intrinsic properties that distinguish our network from 1) a network constructed based on a hypergeometric model alone and 2) a network constructed by randomizing the PIN scores. Another goal was also to evaluate the impact that the bias against membrane proteins would have over the topological properties of the resulting networks. Thus, we selected a cut-off resulting in 5,000 interactions for each network. We denote these sub-networks by adding the suffix -5K, (e.g., PIN-5K). As expected, PIN-5K showed a higher fraction of membrane proteins after considering the total number of nodes in the network: PIN-5K (21%, 639/2999), PIN with random scores-5K (18.5%, 527/2843), and the hypergeometric model-5K (19%, 519/2723).

To evaluate the potential to form hubs, we calculated the probability of a node having a specific number of interactions as the number of nodes in a given degree interval divided by the total number of nodes in the network. We observed that PIN-5K showed lower probability to form hubs (arbitrarily defined as nodes that have more than 10 interactions) than the other two networks. Only 56 (1.87%) of the PIN-5K nodes were hubs, while for PIN with random scores-5K and the hypergeometric model-5K networks 140 (4.92%) and 70 (2.57%) of the nodes were hubs, respectively. Unlike PIN-5K, the hypergeometric model-5K network showed enrichment of hub genes related to RNA processing (p = 6 × 10^-7^), macromolecule biosynthetic process (p = 3 × 10^-7^) and mRNA polyadenylation (p = 3 × 10^-10^), all genes associated with false positives (Additional file 3). Consistent with this interpretation, most functional categories with a difference in enrichment between these networks are those related to genes localized in the nucleus, which is the main cell compartment with over-representation in BIOGRID. As expected, the enrichment p-values observed with the hubs in PIN-5K were consistently higher (lower significance) when compared to what we observed with hypergeometric model-5K network. Together these results suggest that the process of removing bias against membrane proteins resulted in decreased over-representation of some unrelated gene categories, as discussed above.

Having detected a different functional composition in the networks, we next evaluated some network properties that could be affected by removing the bias against membrane proteins. Thus, for each node we calculated the clustering coefficient C_w_, which is defined as the number of interactions between the neighbours of a single node divided by those that could possibly exist. This coefficient is related to the existence of functional clusters [40]. We observed that even though PIN-5K contains fewer hubs, it is more clustered (C_w_ = 0.170) than either the randomized network (C_w_ = 0.006) or the hypergeometric model network (C_w_ = 0.165). For the top 5000 interactions from each network, the lower number of hubs together with the higher clustering coefficient, observed for the PIN suggests a process where the interactions were more equally distributed among the genes, resulting both in increased clustering and decreased average degree.

### Membrane interactions and the centrality-lethality rule

The centrality-lethality rule (CLR) states that there is a correlation between degree (number of interactions/protein) and the essentiality of the corresponding gene [41]. Thus, proteins and genes highly connected with other partners tend to be essential and therefore some of their biological characteristics could be explained by topological features [42,43]. However, He & Zhang [42] suggested that the CLR is unrelated to highly connected genes, but could be explained by the fact that hubs have large numbers of interactions, and thus they have higher tendency to engage in essential interactions. Ivanic et al [44] showed that PPI networks constructed by using interactions from affinity purification procedures have good correlation between degree and abundance while Y2H PPI networks do not. According to the authors, if there is a degree/abundance correlation, there is also a degree/essentiality relationship since the correlation between essentiality and abundance is well established. However, the relationship between essentiality and hubs could be artificial due to essential proteins generally being more abundant [44], and therefore methods biased toward abundant proteins would be artificially inflated with interactions involving essential proteins. In fact, Batada et al [45] reported that the Y2H dataset from Ito et al [12] has a weak correlation between degree and essentiality. This same dataset was analyzed elsewhere, where no difference between degree distributions of essential and nonessential proteins was observed [44]. As we noted previously, membrane proteins tend to be expressed at low levels, and thus we hypothesized that the process of removing bias against membrane proteins could also affect the relationship between degree and essentiality, thus affecting the foundations of the CLR.

**Figure 6:**
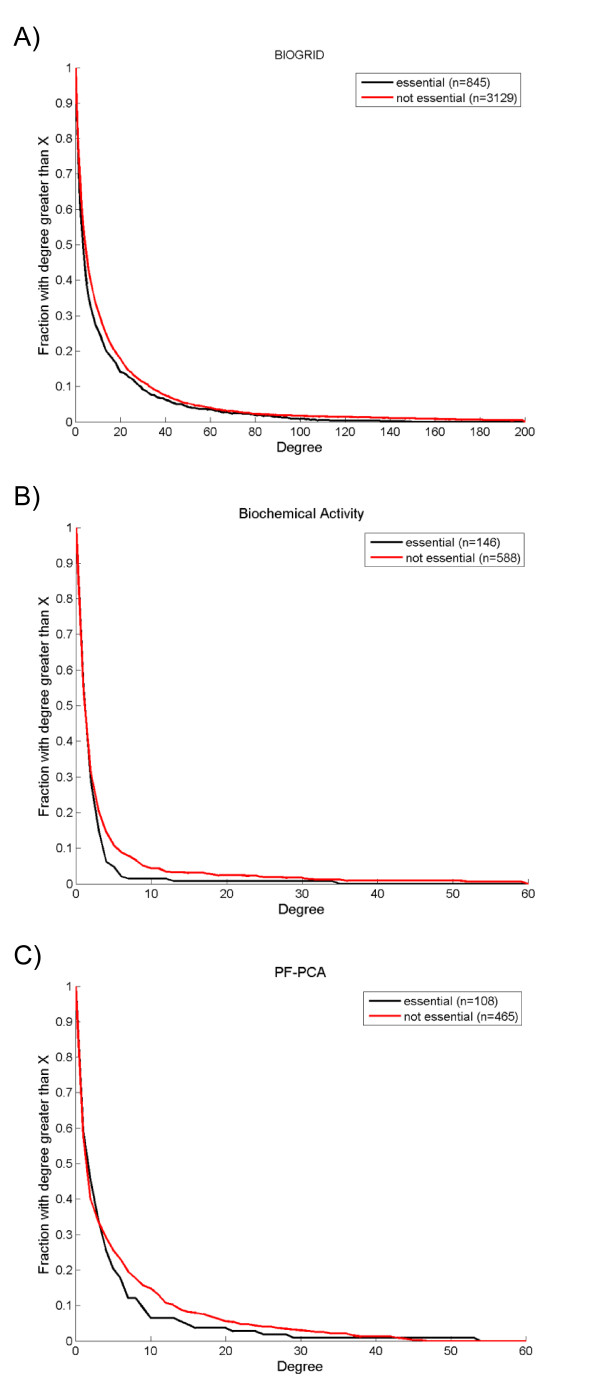
**Contrary to the centrality-lethality rule, the distribution of degree for membrane associated genes is similar or higher for non-essential compared to essential nodes**. Membrane associated genes were identified in each dataset by Gene Ontology and divided into essential (black) and not-essential (red) gene sets. Each plot shows the cumulative distribution of node degree for a network constructed using interaction data from the technique specified. A) BIOGRID, B) Biochemical Activity, C) PF-PCA. Between parentheses is the number of nodes.

To test the hypothesis that the membrane interaction content of a network affects the CLR, we downloaded the list of essential genes from the Saccharomyces Genome Deletion Project [46]. Then we visually compared the degree distributions of essential and nonessential membrane proteins to determine if the degrees are drawn from the same underlying population. We observed different degree distributions of essential and nonessential genes that varied with method used to generate the data. Interestingly, we also observed that the membrane associated genes did not support the CLR (Figure 6A-C).

**Figure 7:**
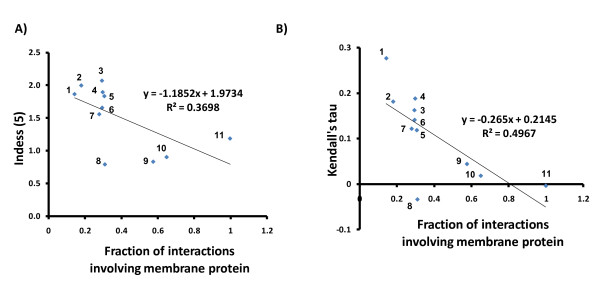
**Membrane associated gene interaction data does not support the centrality-lethality rule**. A) The Indess (5), an *ad hoc *indicator of the centrality-lethality rule, is negatively correlated with the content of membrane interactions (Spearman correlation = -0.72, p = 0.012). Each data point represents a different PPI technique. B) The correlation between Kendall's tau, another *ad hoc *indicator of the centrality-lethality rule, and the content of membrane interactions using different techniques to generate interaction data (Spearman correlation = -0.83, p = 0.003). 1) Affinity capture - MS, 2) Colocalization, 3) Reconstituted complex, 4) Affinity capture - Western, 5) Affintiy capture - RNA, 6) Two hybrid, 7) Copurification, 8) Bioactivity, 9) Cofractionation, 10) PF-PCA, 11) SU-2HY.

To evaluate the relationship between membrane content and the CLR, we developed a nonparametric measure, the Index of Degree and Essentiality (Indess). The Indess(k) of a network is the fraction of the essential genes with degree greater than k divided by the fraction of the non-essential genes with degree greater than k. We applied this measure of association to assess the correlation between degree and essentiality over the networks obtained from all PPI techniques with more than 300 unique interactions available in BIOGRID. Interestingly, when we arbitrarily used k = 5, we observed a negative Spearman correlation (-0.72, p = 0.012) between Indess(5) and the fraction of membrane proteins interacting in the network, suggesting a negative correlation of the CLR and the content of membrane interactions (Figure 7A). Consistent with this interpretation, the Indess(k) showed a negative correlation with the membrane content independent of k (Additional file 2, Figure S5).

We also applied another nonparametric measure of association, the Kendall's tau rank correlation coefficient [43], to assess the relationship between degree and essentiality. This analysis also showed a negative correlation between the CLR and the content of membrane interactions in the network, suggesting that the CLR is valid mainly for interaction datasets with a lower coverage of membrane PPIs (Figure 7B). Thus, the most frequently used PPI technique with the lowest coverage of membrane interactions, affinity capture-MS, showed a strong correlation between degree and essentiality as measured by the Kendall's tau (0.277, p = 9.46E-96) and Indess (1.864), while PF-PCA and SU-2HY, two datasets enriched in membrane interactions, showed no significant correlation between degree and essentiality (Table 2).

**Table 2:** Correlation between membrane interaction content and centrality-lethality rule for different PPI techniques.

Technique	Unique interactions	Fraction of membrane interactions	Kendall's tau	Kendall's tau (p-value)	Indess (5)	Assortativity coefficient
**Affinity Capture-MS**	26350	0.14	0.277	9.46E-96	1.864	-0.071

**Co-localization**	351	0.18	0.181	4.14E-04	1.996	0.214

**Co-purification**	1217	0.28	0.127	1.07E-04	1.558	-0.227

**Reconstituted Complex**	1959	0.29	0.167	3.49E-11	2.069	-0.101

**Two-hybrid (without Miller et al (2005))**	7040	0.29	0.141	9.11E-20	1.657	-0.107

**Affinity Capture-Western**	4953	0.30	0.188	3.92E-24	1.894	0.112

**BIOGRID (all techniques)**	94877	0.30	0.190	4.98E-70	1.312	-0.102

**Affinity Capture-RNA**	1187	0.31	0.119	5.07E-05	1.833	-0.857

**Biochemical Activity**	5355	0.31	-0.034	0.104	0.792	-0.460

**Co-fractionation**	574	0.58	0.044	0.274	0.833	-0.163

**PF-PCA**	2395	0.65	0.018	0.505	0.904	0.171

**SU-2HY**	1941	0.99	-0.004	0.928	1.187	-0.074

Note: the table was sorted in ascending order according the column with the "fraction of membrane interactions".

The Kendall's tau and Indess(5) are both inversely correlated to the content of membrane interactions, while the assortativity coefficient is not.

The analysis above used the PIN generated using an optimized weight for the network enriched in membrane interactions (network A). To further evaluate the impact of our approach on the CLR we calculated the correlation between the weight of network A, and the Kendall's tau and Indess(5). Removal of the bias against membrane proteins showed an impact on the distribution of node degree between essential and nonessential genes (Table 3), reflected in a negative Spearman coefficient between membrane interaction content and the Kendall's tau (-0.90, p = 0.0002) and Indess(5) (-0.78, p = 0.005). These results show that the correlation between high node degree proteins and essentiality that resulted in the CLR is biased due to interaction datasets with an under-representation of membrane proteins.

**Table 3:** Correlation between membrane interaction content and centrality-lethality rule for different weights of network B.

Weight of network A	Fraction of membrane interactions	Kendall's tau	Kendall's tau (p-value)	Indess (5)
**0.0**	0.320	0.23	7.00E-57	4.639

**0.1**	0.322	0.23	1.00E-54	4.701

**0.2**	0.323	0.23	5.55E-55	4.717

**0.3**	0.328	0.23	3.25E-54	4.667

**0.4**	0.328	0.23	1.22E-54	4.740

**0.5**	0.344	0.22	1.55E-51	4.467

**0.6**	0.525	0.13	9.14E-15	1.405

**0.7**	0.721	-0.01	0.663	0.751

**0.8**	0.762	-0.03	0.152	0.784

**0.9**	0.762	-0.03	0.135	0.784

**1.0**	0.764	0.00	0.994	0.953

Note: the table was sorted in ascending order according the column with the "fraction of membrane interactions".

The Kendall's tau and Indess(5) are both inversely correlated to the content of membrane interactions.

Ivanic *et al *[44] found that all yeast PPI datasets contain significant enrichments of essential-essential interactions, suggesting that essential proteins have higher probability of interacting with each other than non-essential proteins have of interacting with either essential or non-essential proteins. If essential proteins have a higher degree than non-essential proteins, we expect to observe more mutually interconnected high-degree hubs than would occur at random, independently of membrane PPI content. To test this, we calculated assortativity coefficients for the data. As assortativity coefficients reflect the correlation between the degrees of all nodes on two opposite ends of a link [47], networks with a positive assortativity coefficient are likely to have mutually interconnected high-degree hubs. On the other hand, networks with a negative assortativity coefficient are likely to have widely distributed high-degree hubs. We observed that most datasets showed negative assortativity coefficients that were not correlated with membrane interaction content (Table 2). Similarly we observed no correlation between membrane interaction content with density, which is the mean network degree, or with mean clustering coefficient (Additional file 2, Table S1). Assuming that essential proteins do have a higher probability of interacting with each other [44], our results suggest that these proteins are not hubs, since our results suggest that hubs don't necessarily interact with each other.

### Analysis of Unfolded Protein Response (UPR) using PIN

All of our data suggest that the PIN is a more robust accurate description of the interactions between proteins and genes in cells than either raw BIOGRID data or BIOGRID data refined using a hypergeometric model. Therefore, we have applied the PIN to the analysis of PPI data for proteins involved in the UPR. In eukaryotic cells, most transmembrane proteins fold and mature in the endoplasmic reticulum (ER). Thus, proteins enter the ER as unfolded polypeptide chains where they acquire their final conformational structure during or after synthesis completes. To manage this process, cells adjust the protein-folding capacity of the ER according to synthetic requirements, thereby ensuring that the quality of cell-surface and secreted proteins can be maintained. The UPR is a eukaryotic stress response initiated by detection of unfolded proteins in the lumen of the ER, and triggers a compensatory response mediated by a large number of co-regulated genes. As the protein folding events involved occur in the ER, the cellular response therefore involves mostly biological processes related to proteins located in the membrane/ER [48].

Thus, we reasoned that our PIN approach should recover biological processes occurring in the UPR more accurately than a probabilistic network constructed without reduction of bias against membrane proteins. In order to verify this hypothesis, we evaluated the fraction of GO Slim terms interacting with 243 UPR genes downloaded from Jonikas et al [49] by using three different networks: 1) PIN, 2) a network constructed using BIOGRID data and the hypergeometric model, which is actually a probabilistic network constructed without removing the bias against membrane proteins, and 3) a network constructed with raw data from the BIOGRID.

**Figure 8:**
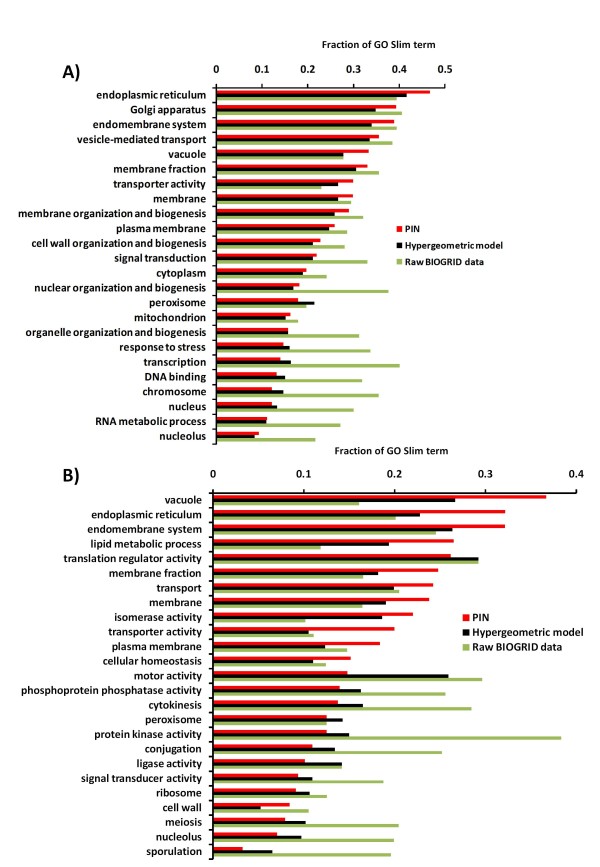
**Analysis of coverage of genes involved in unfolded protein response (UPR) and autophagy in PIN compared to hypergeometric and raw BIOGRID data**. For the 13 GO Slim terms with the highest and lowest interactions with UPR or ATG genes in PIN, the fraction of GO Slim terms was compared for each network. We used the top 20% interactions in each network. A) The distribution of GO Slim terms suggests that for the UPR network, PIN has many fewer false positives than raw BIOGRID data (e.g. terms related to nucleus). B) The distribution of GO Slim terms suggests that for the ATG network PIN has many more true positives than the other methods and less false negatives than the raw BIOGRID data.

We observed that our model showed a better performance when compared to the hypergeometric model for UPR-related GO Slim Terms. This can be visualized in Figure 8A, where for the least 13 covered GO Slim terms those more unlikely to be related to UPR were globally under-represented in our PIN. For example, genes located in the nucleus or related to processes occurring in the nucleus (transcription, DNA binding, etc) were under-represented. On the other hand, some terms relatively more likely to participate in UPR showed a tendency to be better represented (e.g. endoplasmic reticulum, transporter activity and vacuole) in the most 13 covered GO Slim terms. Although other terms that might be involved in UPR have a slightly increased representation in the network constructed with BIOGRID raw data (e.g. vesicle-mediated transport, membrane fraction), it is likely that this is due to the trade-off between sensitivity and specificity. As a result of the low specificity of the raw BIOGRID data, it ends up selecting more true positives at the expense of a much higher number of false positives.

Although there are nuclear genes involved in UPR, they constitute a small fraction of the total UPR related genes and therefore, nuclear genes could be taken as negative standard controls. The slightly lower relative prevalence of interactions involving these categories and UPR genes in PIN indicates that although our network has a higher true positive rate (bona fide UPR interactions) compared to the hypergeometric network, this did not correspond to a higher false negative rate as represented by the coverage of nuclear GO Slim Terms. Thus, these results suggest globally that for UPR related genes our approach was powerful in reducing the false positive rate without unduly sacrificing sensitivity as evidenced by a higher coverage of interactions with GO Slim terms related to UPR processes.

The 3 global networks have the same number of interactions, although they have been scored differently. After selecting the 243 UPR genes and their neighbors in PIN and in the hypergeometric network in which the bias against membrane proteins was not removed, we observed that our model showed more interactions: 2956 interactions (1127 nodes, 2.62 interactions/node) compared to 2359 interactions (1074 nodes, 2.20 interactions/node) from the hypergeometric network. In comparison, the raw data from the BIOGRID resulted in a network with 7049 interactions (1352 nodes, 5.21 interactions/node) consistent with this network containing a very large number of false positives (Figure 8A). The PIN interactions for the UPR network are available as a Cytoscape file in Additional file 4.

Our results suggest that the bias against membrane proteins seems to be an effect derived mainly from physical interactions, since after calculating the correlation between the fractions of GO Slim terms interacting with UPR genes through genetic and physical interactions (the bars in Figure 8A), we observed a low correlation between the fractions identified by PIN and BIOGRID raw data (Pearson's correlation = 0.09). On the other hand, the correlation was very high (0.88) when using only genetic interactions. We presume this effect is because genetic interaction data is not as biased against membrane proteins.

We also evaluated the distribution of PPIs retrieved by the 243 UPR gene set in the PIN, hypergeometric model and BIOGRID networks and observed 47, 26, and 21 interactions in the top 20 K interactions, respectively. Additionally, we evaluated the predictive power of PIN by using the positive reference set (PRS) and random reference set (RRS) described above. In fact, PIN was superior as it retrieved 9 UPR interactions, while the hypergeometric model and BIOGRID retrieved 4 and 0 interactions in the PRS, respectively. On the other hand, the RRS had 2, 16, and 18 interactions retrieved by PIN, hypergeometric model, and BIOGRID, respectively, confirming the lower rate of false positives of PIN. The PRS and RRS sets were not used to construct the networks and therefore, constituted an independent test set.

### Analysis of Autophagy using PIN

Autophagy is a fundamental and phylogenetically conserved cellular quality control process by which cytoplasmic constituents including proteins, protein aggregates, organelles, and invading pathogens can be delivered to lysosomes for degradation. It is characterized by the formation of double-layered vesicles (autophagosomes) around intracellular cargo for delivery to lysosomes and proteolytic degradation. This process occurs as a response to changes in the internal status of the cell and/or changes in the extracellular environment [50,51]. In addition to its essential role for cell survival under nutrient-deprived conditions, autophagy is also involved in a wide range of physiological and pathological processes in eukaryotic organisms [52]. Using the same approach as for UPR, we observed a similar performance when we evaluated PIN using bona fide interactions related to autophagy (Figure 8B), those interactions involving genes that are probable to be functionally involved in the UPR process, that is, interactions involving genes in vacuole, endoplasmic reticulum, etc. The fraction of the GO Slim term interacting with autophagy genes was calculated using the top 20% interactions. Thus, the dramatic over-representation in the uncorrected BIOGRID network by GO Slim terms likely due to the inclusion of false-positives was greatly reduced in the autophagy PIN. Furthermore, for autophagy related genes the PIN clearly outperformed the hypergeometric model in capturing relevant GO Slim terms.

To apply our PIN to overview the network organization of the autophagy system in yeast it was necessary to increase the number of genes annotated as related to autophagy, as only 31 autophagy (ATG) genes have been reported in yeast so far [52]. Thus, we downloaded the human autophagy network with 751 interactions (429 proteins) [53]. This physical interaction dataset was generated by proteomics analysis of mass spectrometry (LC-MS/MS) data of proteins interacting with 32 human proteins linked to autophagy or vesicle trafficking. Using this set of 429 proteins, we found in the INPARANOID database [54] a set of 116 yeast orthologs, which with the addition of 31 yeast genes annotated as ATG-related, was expanded in our PIN, by including their neighbors. The resulting yeast autophagy network encompassed 2316 interactions and 982 genes, whose interactions are available as Cytoscape file in Additional file 4.

**Figure 9:**
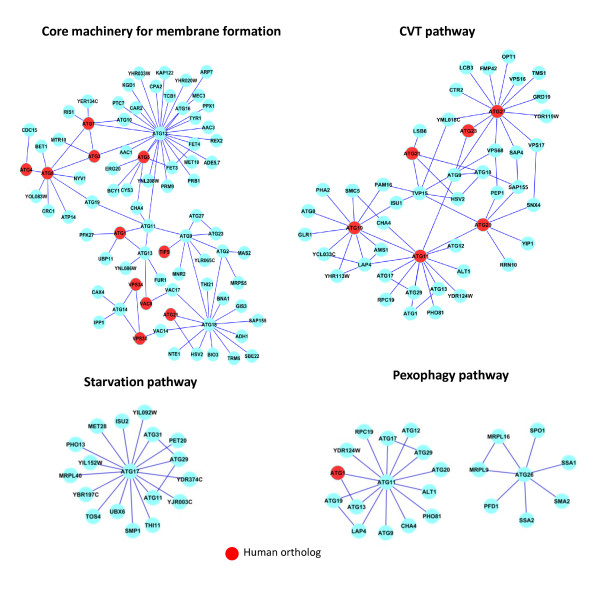
**Autophagy networks identified in PIN**. The nodes (cyan, red) were spread manually to ease visualization and identified with the corresponding SGD gene symbol. Yeast genes with identified corresponding human orthologs (red).

To examine the resulting network, we selected the 4 main autophagy-related pathways; starvation-induced autophagy, the cytoplasm-to-vacuole targeting (CVT) pathway, pexophagy (an autophagic degradation pathway for peroxisomes in yeast) and the core machinery for membrane formation [52]. These pathways are shown in Figure 9 where the genes that have orthologs in the human autophagy network are shown in red. As expected, among the 31 yeast autophagy proteins, the function of Atg17, Atg29, and Atg31 are required specifically for starvation-induced autophagy and they form a ternary complex (Figure 9, the starvation pathway).

We observed that ATG12 was present in the periphery of both the CVT and pexophagy pathways but was absent from the starvation pathway. This result was surprising as while Atg12 is not essential for growth, it is essential for autophagy and for maintaining viability during starvation [55] and it occupies a central role in the core autophagy network by connecting several other ATG genes (ATG3, ATG7, ATG5, ATG16, ATG11, and ATG10). Atg12 is part of two protein conjugation systems, each composed of two ubiquitin-like proteins (Atg8 and Atg12) and three enzymes (Atg3, Atg7 and Atg10 shown in Figure 9, the core pathway) that are required for their conjugation reactions. Atg12 forms a conjugate with Atg5, whereas Atg8 is conjugated to phosphatidyl ethanolamine, a major component of various biological membranes. Both conjugates localize to autophagy-related membranes, suggesting their direct involvement in the biogenesis of these membranes [52].

Interestingly, the Atg17-Atg29-Atg31 complex proposed to function as a conductor together with the Atg1-Atg13 complex in organizing the pre-autophagosomal structure [52], formed a cohort of genes with ATG11 acting as a hub (Figure 9, the pexophagy pathway). Although we used the pexophagy-associated genes ATG11, ATG25, ATG28, ATG30, and ATG26 plus their neighbors to select the interactions representing pexophagy in our network, we only retrieved the genes interacting with ATG11 and ATG26 from PIN. The other 3 genes (ATG25, ATG28, and ATG30) were not present in our autophagy network. Consistent with our network analysis, all these 3 genes showed inconsistent nomenclature when we searched the SGD database [56].

To better organize the information available in the yeast autophagy network and to reveal larger interacting functional modules, we applied a recently developed algorithm based on link communities [57]. This algorithm performs a hierarchical clustering of interactions, that is, instead of assuming that a functional module is a set of nodes with many interactions between them, it considers a module to be a set of closely interrelated interactions. In contrast to the existing algorithms, which have entirely focused on grouping nodes, resulting in modules without overlap, by performing clustering at the level of interactions instead of genes this algorithm naturally incorporates imbricate sets while revealing hierarchical organization in the network. Interestingly, and consistent with the data in Figure 9, in this analysis we observed that the starvation pathway is a distinct set of interactions with low overlap with the other three autophagy processes examined here (Additional file 2 Figure S6). In fact, it was shown elsewhere that portions of the endoplasmic reticulum can be selectively packaged into autophagosomes upon induction of the UPR or during starvation. Under strong UPR-inducing conditions, ER-phagy depends on Atg proteins. However, when induced by starvation, ER-phagy is only partly Atg-dependent. This suggests that during starvation, selective uptake of portions of the ER by autophagosomes may not use the entire phagophore assembly machinery [58]. This is consistent with our network observations showing that the starvation pathway constitutes a relatively distinct pathway among the ATG interactions.

## Discussion

By integrating diverse sources of data based on physical and genetic interactions, we constructed a probabilistic network that can be used to analyze biological processes occurring in the membranes of the yeast *Saccharomyces cerevisiae*. The analysis of interactions involving membrane proteins poses several challenges that are distinct from those encountered for soluble proteins. For example, the expression level of membrane proteins is lower compared to soluble proteins [19]. Thus, any network approach with the goal of good predictive power needs to be re-assessed for membrane proteins.

Here we applied a statistical model combining hypergeometric distribution and logistic regression to increase the coverage of membrane proteins, using the BIOGRID database as a reference. In this combined model, the hypergeometric distribution model was used to construct two networks with the most statistically significant interactions, while logistic regression was used to optimally combine these two networks to increase the coverage of membrane proteins without sacrificing prediction performance. Our results reinforce the necessity of considering coverage and prediction performance together in the process of constructing probabilistic networks, since an interaction dataset of high coverage is compromised if it contains many false positives (low prediction performance). The prevalence of false positive interactions is suggested by the fact that despite numerous analyses about 41% of yeast PPIs exist in only one database [59]. By increasing the score threshold to identify and to remove non-specific interactions and by replacing lower quality interactions with higher quality membrane associated interactions our PIN resulted in a more robust network for all interactions which can be used to analyze a variety of biological data. Our approach also allows the raw estimation of limits for prediction performance and coverage (see Figures 3 and 4) of membrane interactions, revealing the biased characteristics of publicly available interaction data. The effectiveness of this approach was shown by the increased coverage of membrane proteins in PIN and by the comparable predictive performance relative to the hypergeometric model alone.

In a similar approach, Xia *et al. *[6] integrated genomic features (genome-wide sequence, function, localization, abundance, regulation, and phenotype data in yeast) to predict yeast helical membrane protein interactions using a logistic regression classifier. Also, Zhang & Ouellette [60] proposed a method to predict interactions between membrane proteins using a probabilistic model based on the topology of protein-protein interaction network and that of domain-domain interaction network in yeast. Here, we used a hypergeometric model plus logistic regression to select interactions in BIOGRID in a manner which effectively reduced the bias against membrane proteins in the resulting network. Although these other approaches also used logistic regression to represent interactions involving membrane proteins more accurately, their approach is a prediction algorithm designed to identify a particular kind of membrane protein interactions using training sets of interaction data [6,60]. In contrast, we have constructed a PIN that allows the construction of less biased networks with the goal of analyzing all membrane proteins.

The PIN we constructed provided new insight into the applicability of the CLR (centrality-lethality rule) to membrane proteins, provided a more balanced view of the UPR (unfolded protein response) and confirmed the modular nature of the autophagy network while simultaneously expanding the number of PPIs predicted to be involved in this crucial cellular process in yeast. Our approach can be adapted to other contexts to increase the coverage of specific classes of PPIs. For example, a network could be constructed specifically to model interactions occurring in mitochondria, which is also a category under-represented in BIOGRID (Table 1). However, for each such network, it is necessary to first choose an adequate positive gold standard of proteins or interactions in order to optimize parameters and maximize prediction performance and coverage of the specific class of interactions.

Our analysis suggests that some topological properties of the yeast interactome can also be biased due to the over-representation of proteins expressed in higher levels, which are often essential proteins, and to the parallel low representation of membrane PPIs. Although not directly related to the lethality-essentiality rule, the scale-free model of biological networks is often used to explain the robustness of these networks. However, this explanation is made by assuming that highly connected proteins tend to be essential and taking for granted that proteins have widely different connectivities. We observed in this study that part of this unequal distribution of degrees can be due to the bias towards abundant proteins and, by extension, against membrane proteins. In fact, our findings could help to explain why scale-free topology of some partial interactome data cannot be confidently extrapolated to complete interactomes [61]. In addition, it was also noted the discrepancies in matching existing interactome networks to a scale-free topology, suggesting that the structure of PPI networks is better modeled by a geometric random graph than by a scale-free model [62].

## Conclusions

Our results suggest that selection against membrane protein interactions is a key factor determining the prediction performance of PPI networks in yeast and probably for other organisms. We demonstrate that this bias affects topological properties of the resulting probabilistic network. In addition, our findings also suggest that the validity of the CLR, and other factors associated with PPI networks, such as coverage of some interactions occurring in membrane-associated compartments, are likely to be affected by these constraints. Our simple computational approach was effective in decreasing this bias and allowed the construction of a probabilistic network that is more representative of the real interactions occurring in yeast. Our PIN allowed us to make new predictions about the regulation of UPR and autophagy, two essential processes associated with subcellular membranes.

## Methods

### Construction of PIN using the guilty-by-association approach

PIN was constructed in seven steps:

1) Calculate the significance of each interaction in BIOGRID using hypergeometric distribution (see below) in order to construct networks A and B (see paper). This should be done separately for each network A and B.

2) Sum up the scores for redundant interactions in each network A and B.

3) Combine networks A and B using logistic regression (see below) with different weights. This step generates 10 networks with weights of network A varying between 0 and 1.

Training the 10 networks:

4) Divide the GO Slim terms between membrane and non-membrane annotations. Train the network against the training set of all genes-GO Slim annotations by selecting two thirds of the pairs gene-GO Slim annotation. These pairs will be used for training the network. The rest is the test set. The training is done by summing up the scores associated with the pairs gene-GO Slim annotation used as training set, e.g., if a gene is associated in the network to 3 membrane genes, the scores of these 3 interactions are summed up. These scores are the -log10(p-value) of the hypergeometric distribution calculated in the step 1, which evaluates the significance of interactions, that is, those most significant interactions have a lower p-value (higher score).

Please note that there are two scores: one for the significance of interactions (from the hypergeometric distribution) and the other for the association with membrane genes, which is calculated by summing up these scores (from the hypergeometric distribution) of all membrane genes associated with that particular gene. Thus, the more membrane genes a gene is associated with, the higher the score (for membrane localization) for that gene.

5) Once the network is trained, that is, after each gene has a score associated to the probability of being membrane, obtain the true labels (membrane or non-membrane) for each gene in test set by using the test set (from step 4) of GO Slim annotations.

6) Calculate the AUC of precision-recall curves. To do that, it's necessary two vectors: one with the binary values corresponding to true positives and false positives (1 or 0, respectively) generated in the step 5, the other vector contains the respective prediction scores.

7) Select one network (out of 10) with the best tradeoff between membrane content and AUC.

### Hypergeometric distribution

We applied a probabilistic approach to construct a network with less bias against membrane proteins by using a simple and general error model based on the hypergeometric distribution to calculate the probability for each interaction occurring at random [32]. This score system has proved to be robust across different settings. It penalizes high-degree "promiscuous" interactors since the probability of occurrence of an interaction with this type of partner is high in a random network. In other words, this model penalizes proteins interacting with many different partners because these interactions have a high probability of occurring by random chance, indicating strongly context-dependent interactions. Thus, such cases receive a proportionally low score in the network.

Given an interaction dataset, the probability that two genes A and B interact with each other at random is:

$$-\log P=-\log\left(\overset{\min\;\left(n,m\right)}{\underset{i=k}{\sum }}p\left(i|n,m,N\right)\right)$$

Where:

$$p\left(i|n,m,N\right)=\frac{\left(\begin{array}{c} n\\ i \end{array}\right)\left(\begin{array}{c} N-n\\ m-i \end{array}\right)}{\left(\begin{array}{c} N\\ m \end{array}\right)}$$

In this approach, P indicates the probability that genes A and B will interact by chance. For each interaction:

*n *and *m *= number of interactions in which each gene A and B is involved, respectively

*N *= total number of interactions observed in the entire dataset

*k *= the number of times the interaction between A and B is observed

We applied this hypergeometric distribution model to construct two networks: network A, constructed based on interaction datasets PF-PCA and SU-2HY, and network B, constructed with data from the BIOGRID database (version 2.0.57) without those two datasets. Network A is enriched in interactions involving membrane proteins.

### Logistic regression

To combine the two networks (A and B) in such way that the coverage of membrane PPIs was increased while maintaining the prediction performance, we used a simple logistic regression model with one free parameter to combine the scores of networks A and B by optimizing a precision versus recall performance curve [31]. Thus, for a given gene *i *and a target function *j*, let the probability score computed by the networks A and B be equal to P_*X*_(*i,j*) and P_*Y*_(*i,j*), respectively. Then we can calculate the corresponding combined score P(*i,j*) defined as:

$$\log\left(\frac{P\left(i,j\right)}{1-P\left(i,j\right)}\right)=w.\log\left(\frac{Px\left(i,j\right)}{1-Px\left(i,j\right)}\right)+\left(1-w\right).\log\left(\frac{p_{y}\left(i,j\right)}{1-P_{y}\left(i,j\right)}\right)$$

Logistic regression was applied to combine the networks with the optimal weight parameter *w*. The factor *w *was optimized over the range from 0 to 1 (window = 0.1) to produce the highest area under the precision versus recall curve using as reference genes annotated as membrane protein (GO:0016020). This produced 10 networks, each one using different weights for networks A and B. The best weight (out of 10) was selected based on the guity-by-association approach using the Precision-Recall curve and membrane interactions as reference.

### Analysis of GO enrichment

To analyze the enrichment of GO categories in each level of significance of the PIN, we ranked the network in decreasing order of scores and divided the interactions into quintiles. We then randomly sampled 1000 interactions in each quintile and selected the unique set of ORFs to perform a GO enrichment analysis using BINGO [63]. Categories with enrichment more significant than p < 0.001 after Bonferroni's correction were selected to be plotted.

### Validation of the network

To calculate precision and recall, we performed three-fold cross-validation to obtain a probability score for each gene-GO Slim annotation using a guilt-by-association approach, and used these scores to assess performance [31]. Thus, we downloaded GO Slim annotations and used this dataset by randomly selecting 1/3 of the pairs gene-annotation to hold out for the testing set. Therefore, we applied this analysis on one subset (the training set), and validated on the other independent subset (the testing set).

### Network density

To calculate network density (Nden), we applied the formula:

$$\text{Nden}=2K∕\left(N^{2}-N\right);$$

Where: N is the number of nodes and K is number of interactions in the network.

### Index of Degree and Essentiality (Indess)

We defined the Indess(k) of a network as the fraction of the essential genes with degree greater than k divided by the fraction of the non-essential genes with degree greater than k:

$$\text{Indess}\left(k\right)=\frac{e\left(k\right)∕E}{f\left(k\right)∕F}$$

Where:

*e(k) *= number of essential genes with degree greater than k in the network

E = total number of essential genes in the network

*f(k) *= number of non-essential genes with degree greater than k in the network

F = total number of non-essential genes in the network

Thus, an Indess(k) > 1 indicates that essential genes tend to have a higher degree than non-essential genes, while an Indess(k) < 1 suggests essential genes tend to have lower degree, and Indess(k) = 1 that there's no difference between essential and non-essential genes in terms of degree.

### Assortativity coefficient

The assortativity is a correlation coefficient for the degrees of linked nodes. A positive assortativity coefficient indicates that nodes tend to link to other nodes with the same or similar degree [47].

### Link communities

The algorithm based on link communities performs a hierarchical clustering of interactions and considers a module to be a set of closely interrelated interactions. Given the set of node i and its neighbours n_+_(i), for link pairs that share a node k, the similarity between links e_ik_ and e_jk_ is S(e_ik_, e_jk_) = |n_+_(i) ∩ n_+_(j)|/|n_+_(i) U n_+_(j)| [57]. We implemented this algorithm in MatLab. Then we used the GENE-E [64] to perform the average-linkage hierarchical clustering with Kendall's tau as similarity metric distance.

## Authors' contributions

GCB and DWA conceived of the study, participated in its design, performed the statistical and bioinformatics analysis, and wrote the manuscript. Both authors read and approved the final manuscript.

## Supplementary Material

Additional file 1 **PIN network**. PIN interactions with p-values and scores. Click here for file

Additional file 2 **Supplementary figures**. This file contains the following Supplementary figures and table: **Figure S1 - **Network A interactions are distributed evenly across the top 60,000 of the 94,879 interactions in PIN. Coverage of Network A (rich in membrane interactions) is shown for interactions with different cutoffs of PIN. **Figure S2 - Construction of iREF network as a control weighted network**. The iREF network was constructed by the optimized combination of two networks: A' comprised of interactions not previously used from the iREF database and B, the same network B used to construct PIN. The area under the curve (AUC) of Precision-Recall plots for the top 5,000 interactions was used for selecting the optimum weight for network B using membrane proteins from GO Slim as an established benchmark dataset. The AUC was calculated for different weights for network B, and the weight corresponding to prediction performance in the plateau with higher number of membrane interactions (AUC = 0.38) was chosen as the optimal weight of network B (0.8). Like PIN the performance of this network increased with increasing membrane interactions. **Figure S3 - The enrichment analysis of PIN shows over-represented gene categories (red) occur only in the lower significance quintiles**. The analysis was repeated 10 times by randomly resampling 1000 interactions in each different quintile of the network (I-V). The median p-value of the 10 analysis is shown. A) Cell compartment. B) Biological process. C) Molecular function. **Figure S4 - PIN increases the proportion of membrane associated interactions in the network compared to a hypergeometric model or random scoring**. Coverage of interactions by PIN of genes with related GO terms. **A) **membrane complexes (GO:0030119: AP-type membrane coat adaptor complex [17 proteins], GO:0072546: ER membrane protein complex [6 proteins], GO:0005744: mitochondrial inner membrane presequence translocase complex [11 proteins], GO:0005742: mitochondrial outer membrane translocase complex [8 proteins], GO:0030119: AP-type membrane coat adaptor complex [17 proteins], GO:0072379: ER membrane insertion complex [7 proteins], GO:0005744: mitochondrial inner membrane presequence translocase complex [11 proteins]) or B) Transport genes **Figure S5 - Correlation between Indess(k) and membrane content varies with k **Indess(k) for k = 5-100 varies between -0.5 and -0.2 and is always negative. **Figure S6 - Hierarchical clustering using PIN separates starvation induced genes from other autophagy process genes**. The similarity between interactions was used to build a dendrogram where each leaf is a link (interaction) from the original network and branches represent link (interaction) communities. Each row or column represents an interaction in the autophagy network and the values in the matrix are Z-scored similarity distances between interactions. The red squares in the matrix represent modules as sets of closely interrelated interactions. Each square in the matrix was examined for overlap with each ATG process and annotated manually (bars to the right). **Table S1 - **Network density and clustering coefficient are not correlated with the fraction of membrane interactions reported by each technique. Click here for file

Additional file 3 **GO enrichment of hubs**. GO enrichment of hubs (degree > 10) for different networks. Click here for file

Additional file 4 **Cytoscape file**. Cytoscape format file of autophagy and UPR interactions from PIN. Click here for file
