# Supplementary material for: Removing bias against membrane proteins in interaction networks
Source: BMC Syst Biol. 2011 Oct 19;5:169. doi: 10.1186/1752-0509-5-169 (PMC3213014; doi:10.1186/1752-0509-5-169)
Supplement: Additional file 2 — Supplementary figures. This file contains the following Supplementary figures and table: Figure S1 - Network A interactions are distributed evenly across the top 60,000 of the 94,879 interactions in PIN. Coverage of Network A (rich in membrane interactions) is shown for interactions with different cutoffs of PIN. Figure S2 - Construction of iREF network as a control weighted network. The iREF network was constructed by the optimized combination of two networks: A' comprised of interactions not previously used from the iREF database and B, the same network B used to construct PIN. The area under the curve (AUC) of Precision-Recall plots for the top 5,000 interactions was used for selecting the optimum weight for network B using membrane proteins from GO Slim as an established benchmark dataset. The AUC was calculated for different weights for network B, and the weight corresponding to prediction performance in the plateau with higher number of membrane interactions (AUC = 0.38) was chosen as the optimal weight of network B (0.8). Like PIN the performance of this network increased with increasing membrane interactions. Figure S3 - The enrichment analysis of PIN shows over-represented gene categories (red) occur only in the lower significance quintiles. The analysis was repeated 10 times by randomly resampling 1000 interactions in each different quintile of the network (I-V). The median p-value of the 10 analysis is shown. A) Cell compartment. B) Biological process. C) Molecular function. Figure S4 - PIN increases the proportion of membrane associated interactions in the network compared to a hypergeometric model or random scoring. Coverage of interactions by PIN of genes with related GO terms. A) membrane complexes (GO:0030119: AP-type membrane coat adaptor complex [17 proteins], GO:0072546: ER membrane protein complex [6 proteins], GO:0005744: mitochondrial inner membrane presequence translocase complex [11 proteins], GO:0005742: mitochondrial outer membrane transloc [file 1752-0509-5-169-S2.PPT]

## Slide 1
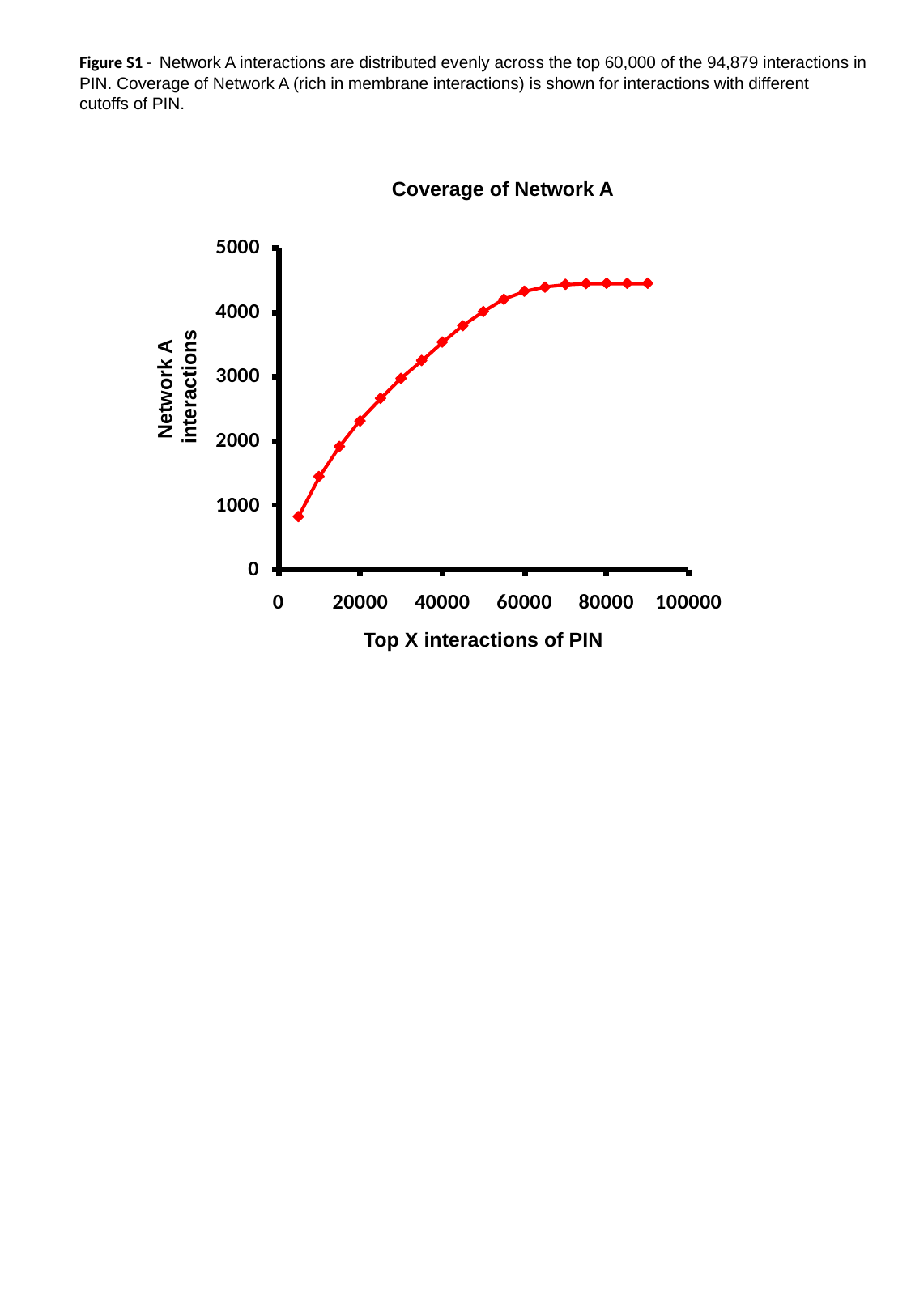

Figure S1 - Network A interactions are distributed evenly across the top 60,000 of the 94,879 interactions in
PIN. Coverage of Network A (rich in membrane interactions) is shown for interactions with different
cutoffs of PIN.
Coverage of Network A
Network A
interactions
Top X interactions of PIN

## Slide 2
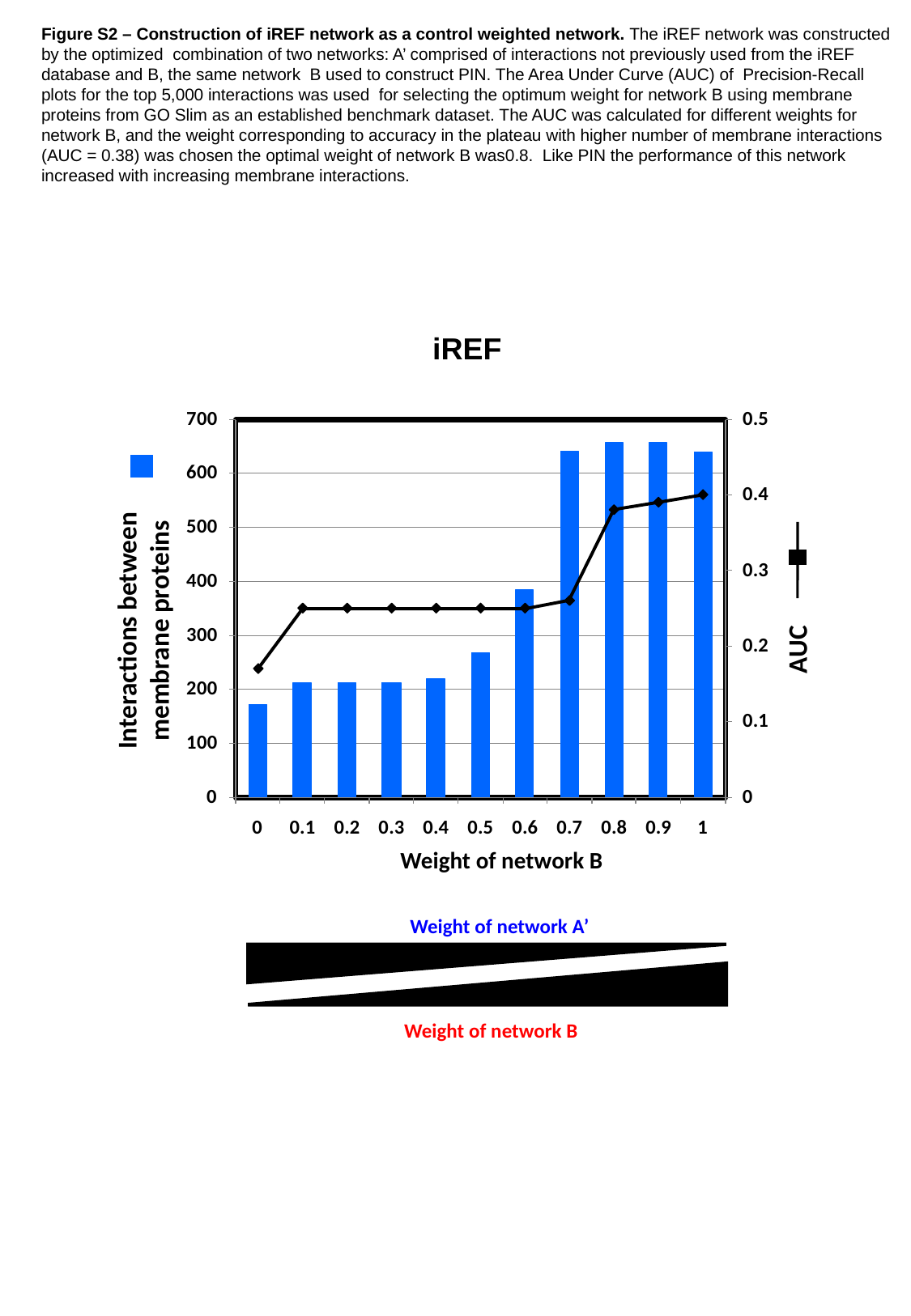

Figure S2 – Construction of iREF network as a control weighted network. The iREF network was constructed
by the optimized combination of two networks: A’ comprised of interactions not previously used from the iREF
database and B, the same network B used to construct PIN. The Area Under Curve (AUC) of Precision-Recall
plots for the top 5,000 interactions was used for selecting the optimum weight for network B using membrane
proteins from GO Slim as an established benchmark dataset. The AUC was calculated for different weights for
network B, and the weight corresponding to accuracy in the plateau with higher number of membrane interactions
(AUC = 0.38) was chosen the optimal weight of network B was0.8. Like PIN the performance of this network
increased with increasing membrane interactions.
iREF
Interactions between
membrane proteins
AUC
Weight of network B
Weight of network A’
Weight of network B

## Slide 3
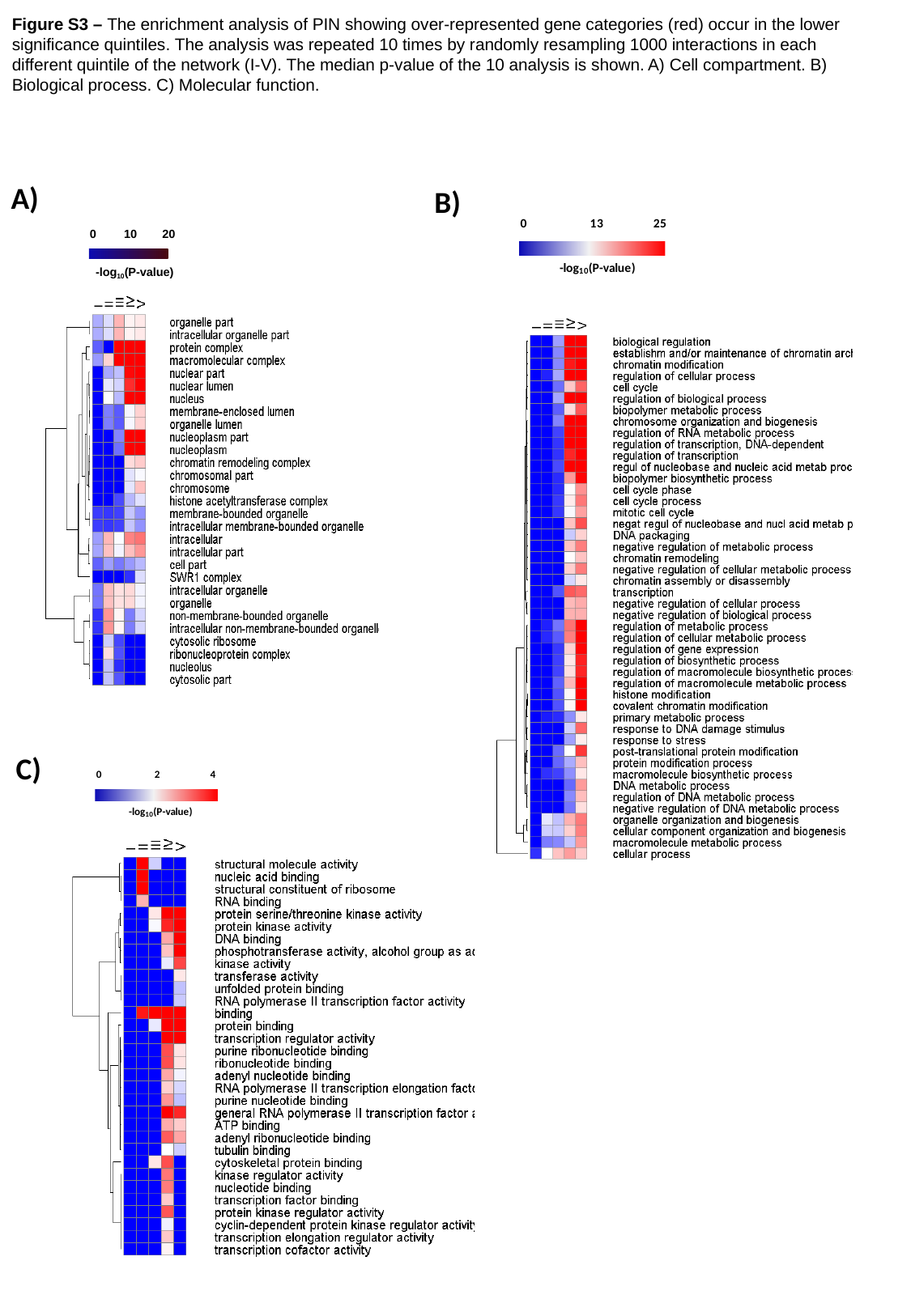

Figure S3 – The enrichment analysis of PIN showing over-represented gene categories (red) occur in the lower significance quintiles. The analysis was repeated 10 times by randomly resampling 1000 interactions in each different quintile of the network (I-V). The median p-value of the 10 analysis is shown. A) Cell compartment. B) Biological process. C) Molecular function.
A)
B)
0
10
20
-log10(P-value)
C)

## Slide 4
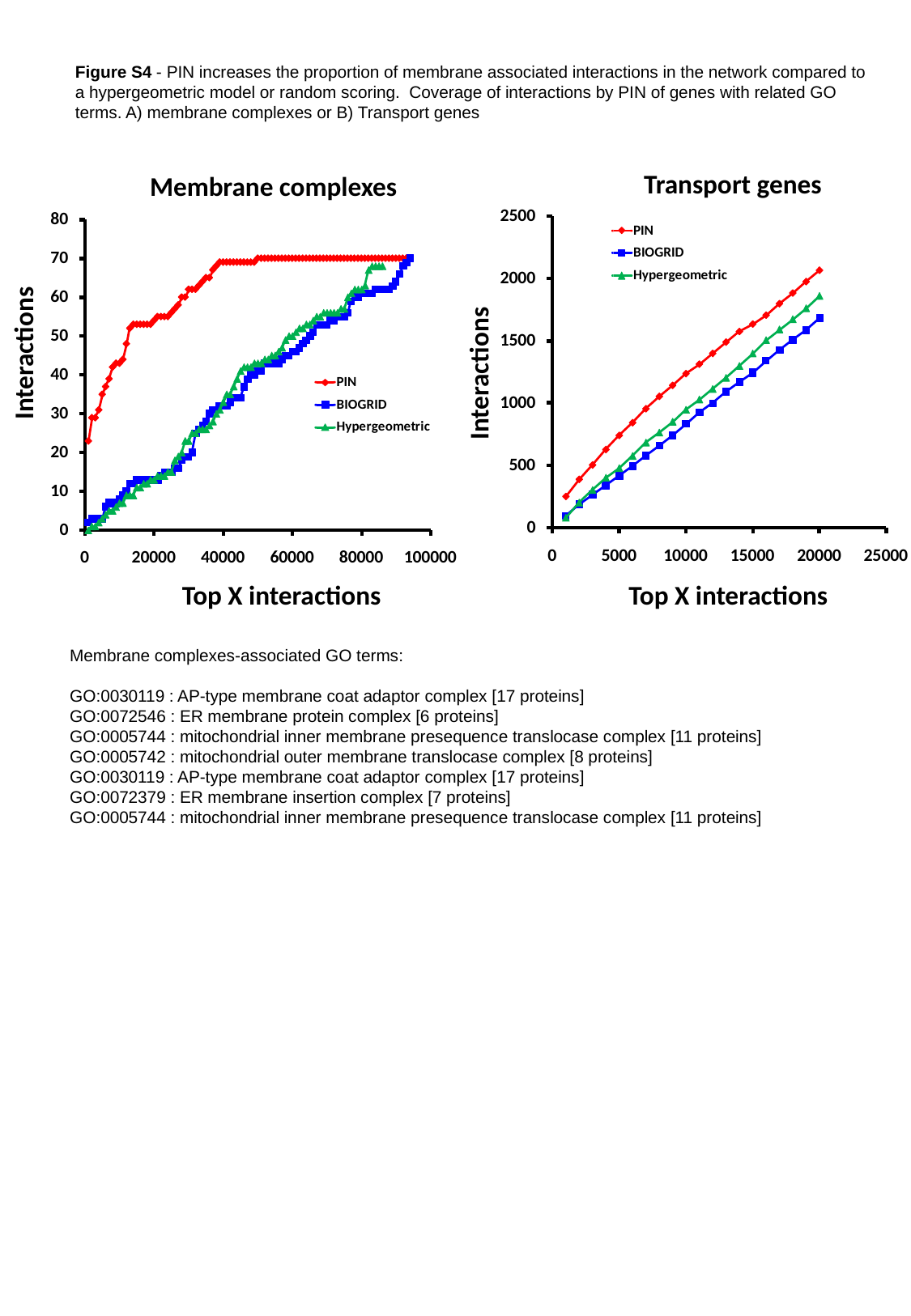

Figure S4 - PIN increases the proportion of membrane associated interactions in the network compared to a hypergeometric model or random scoring. Coverage of interactions by PIN of genes with related GO terms. A) membrane complexes or B) Transport genes
Transport genes
Membrane complexes
 Interactions
 Interactions
Top X interactions
Top X interactions
Membrane complexes-associated GO terms:
GO:0030119 : AP-type membrane coat adaptor complex [17 proteins]
GO:0072546 : ER membrane protein complex [6 proteins]
GO:0005744 : mitochondrial inner membrane presequence translocase complex [11 proteins]
GO:0005742 : mitochondrial outer membrane translocase complex [8 proteins]
GO:0030119 : AP-type membrane coat adaptor complex [17 proteins]
GO:0072379 : ER membrane insertion complex [7 proteins]
GO:0005744 : mitochondrial inner membrane presequence translocase complex [11 proteins]

## Slide 5
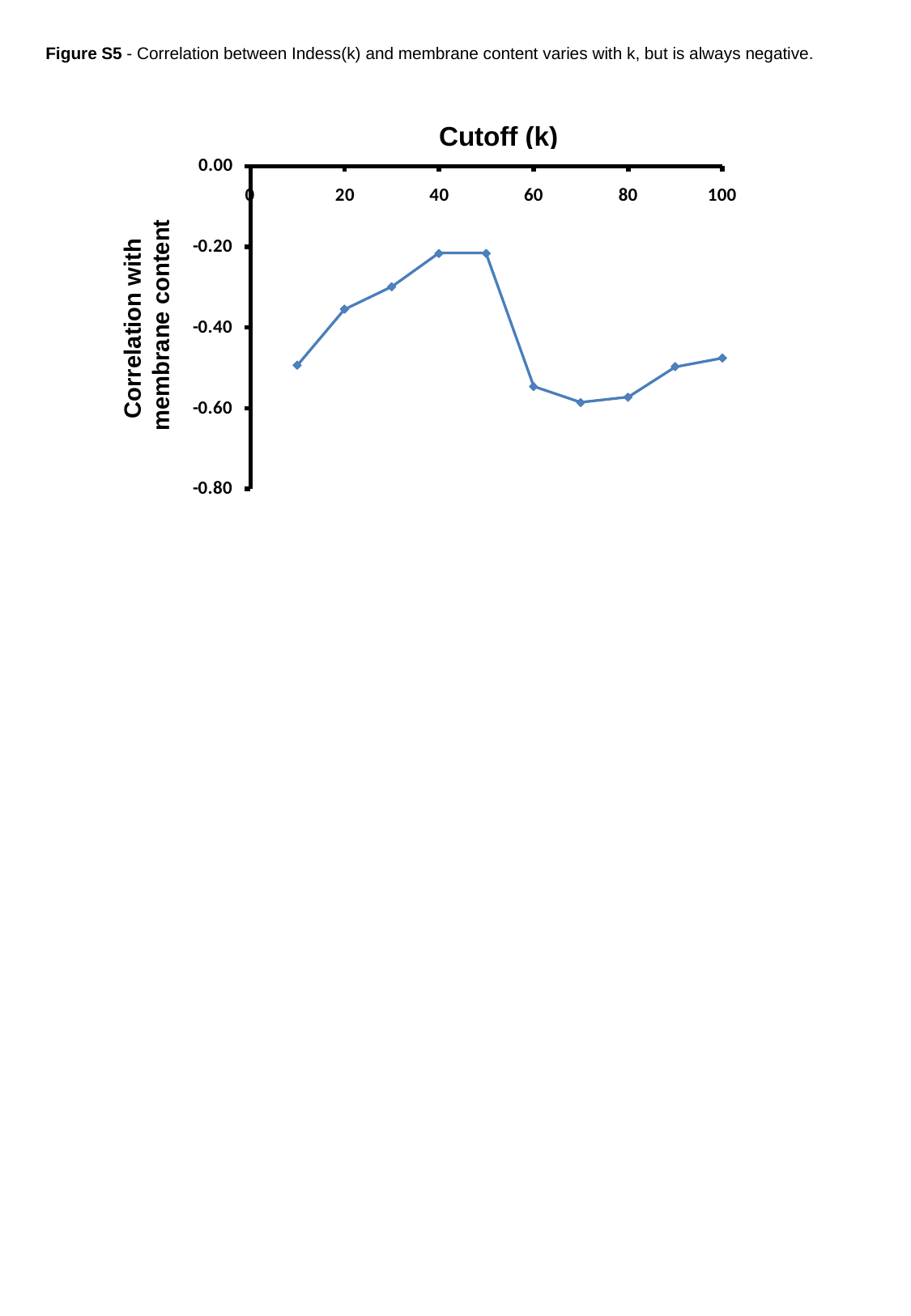

Figure S5 - Correlation between Indess(k) and membrane content varies with k, but is always negative.
Cutoff (k)
Correlation with
membrane content

## Slide 6
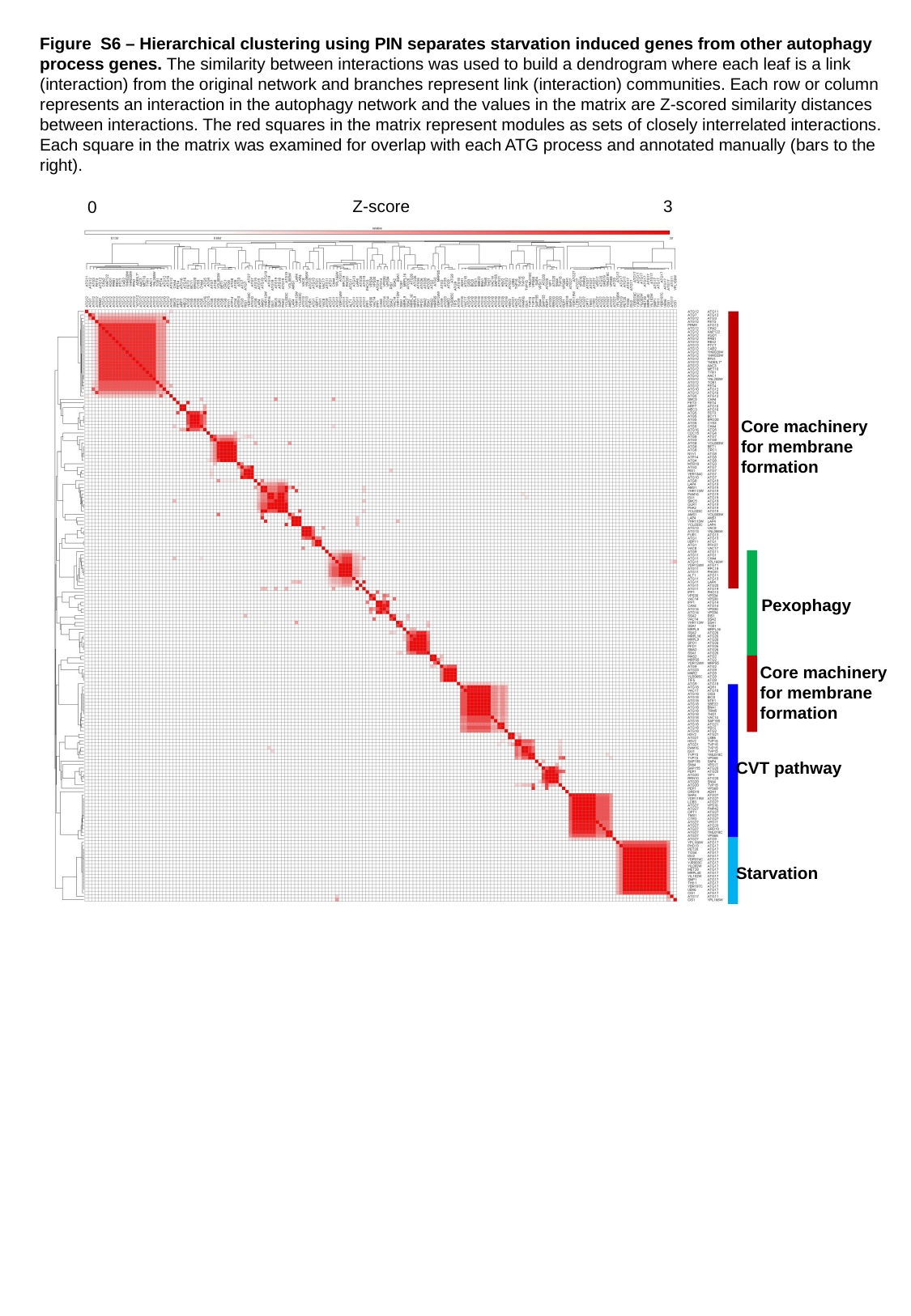

Figure S6 – Hierarchical clustering using PIN separates starvation induced genes from other autophagy
process genes. The similarity between interactions was used to build a dendrogram where each leaf is a link
(interaction) from the original network and branches represent link (interaction) communities. Each row or column
represents an interaction in the autophagy network and the values in the matrix are Z-scored similarity distances
between interactions. The red squares in the matrix represent modules as sets of closely interrelated interactions.
Each square in the matrix was examined for overlap with each ATG process and annotated manually (bars to the
right).
Z-score
3
0
Pexophagy
Core machinery
for membrane
formation
CVT pathway
Starvation
Core machinery
for membrane
formation

## Slide 7
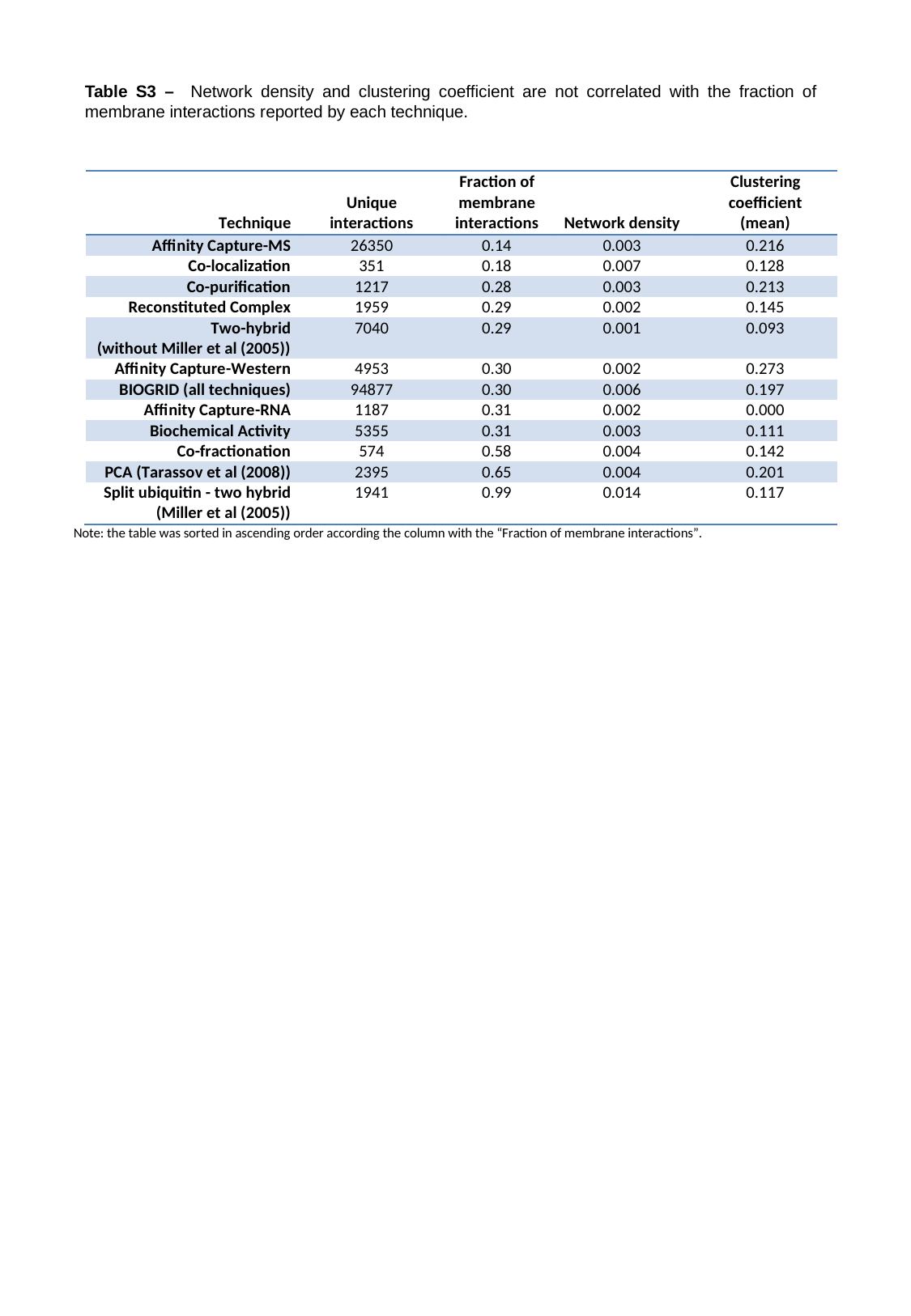

Table S3 – Network density and clustering coefficient are not correlated with the fraction of membrane interactions reported by each technique.
